# The association of n-3 fatty acid intake with muscle mass and strength in older adults: A cross-sectional analysis of the UK biobank data

**DOI:** 10.1016/j.jnha.2025.100622

**Published:** 2025-06-27

**Authors:** Maha Timraz, Marion Guerrero Wyss, Terry J. Quinn, Emilie Combet, Carlos Celis-Morales, Stuart R. Gray

**Affiliations:** a School of Cardiovascular and Metabolic Health, University of Glasgow, Glasgow, United Kingdom; b School of Medicine, Dentistry and Nursing, University of Glasgow, Glasgow, United Kingdom; c Human Performance Lab, Education, Physical Activity, and Health Research Unit, Universidad Católica del Maule, Talca, Chile; d High-Altitude Medicine Research Centre (CEIMA), Universidad Arturo Prat, Iquique, Chile; e Institute of Sports Science and Innovation, Lithuanian Sports University, Lithuania

**Keywords:** n-3 fatty acids, Muscle strength, Muscle mass, Sarcopenia, Older adults

## Abstract

**Objectives:**

The main aim was to investigate the association of n-3 fatty acid intake and the n-6/n-3 fatty acid intake ratio with muscle mass and strength in older adults.

**Methods:**

This study included 61,381 individuals (28,187 men and 33,194 women) aged ≥60 years. Grip strength and muscle mass index were assessed and n-3 and n-6 fatty acid intake were determined. Regression models adjusted for age, deprivation index, ethnicity, month of assessment, total energy intake, multimorbidity, lifestyle factors and physical activity. A sensitivity analysis was conducted in participants aged ≥65 years and in people with sarcopenia.

**Results:**

Data are presented as trend for quintiles from fully adjusted models. Higher n-3 fatty acid intake was associated with a higher grip strength in both men (0.114 kg; 95% CI: 0.02 to 0.21) and women (0.115 kg; 95% CI: 0.05 to 0.18). Similar results were reported for grip strength index, with no associations observed for muscle mass index. No associations were seen in people ≥65 years. In people with sarcopenia no associations of n-3 fatty acid intake with grip strength or grip strength index were seen, but a positive association with muscle mass index was noted in men (0.197 kg/m²; 95% CI: 0.05 to 0.33). The n-6/n-3 fatty acid intake ratio was associated with grip strength in women (0.081 kg; 95% CI: −0.16 to 0.000) and with muscle mass index in men (−0.016 kg/m²; 95% CI: −0.02 to 0.00), no other associations were observed. No associations were seen in people with sarcopenia or people ≥65 years.

**Conclusion:**

Higher n-3 fatty acid intake, with no consistent association with the n-6/n-3 fatty acid intake, was modestly associated with grip strength, with effects varying by sex and age, suggesting limited benefit for sarcopenia prevention at typical intake levels in older adults.

## 1 Introduction

The ageing process is a complex and multifaceted phenomenon marked by numerous physiological changes. Among these changes is a progressive decline in muscle strength and mass, beginning around 30–40 years of age, commonly called sarcopenia [1]. Sarcopenia has emerged as a critical contributor to functional decline and increased risk of chronic diseases in older adults, highlighting the urgent need for effective strategies to mitigate its impact as the global population continues to age [2,3]. It compromises mobility and overall physical performance and exacerbates the risk of developing conditions such as frailty, osteoporosis, and metabolic disorders [2,4]. In the UK alone, the excess health and social care costs linked to muscle weakness are estimated at around £2.5 billion annually [5,6].

Among these potential strategies, nutritional supplementation with n-3 fatty acids has emerged as a promising avenue [[7], [8], [9], [10]]. The primary dietary n-3 fatty acids are eicosapentaenoic acid (EPA) and docosahexaenoic acid (DHA), which are abundant in marine-derived fish oils particularly from oily fish such as salmon, mackerel, sardines, and herring. In contrast, α-linolenic acid (ALA), found in plant-based sources like walnuts, flaxseeds, and chia seeds, undergoes limited conversion to EPA and DHA via elongation and desaturation [11]. There is very little evidence supporting a direct effect of ALA on skeletal muscle health [11], so most of the research in this area has focussed on EPA and DHA. While these marine-derived n-3 fatty acids have long been studied in relation to cardiovascular health [12,13], recent work has investigated potential roles in muscle health in older adults [12,14]. For example, fish oil-derived n-3 fatty acids have been shown to enhance muscle protein synthesis during a hyperaminoacidemic-hyperinsulinemic clamp in middle-aged and older adults, with no effect on basal muscle protein synthesis [13,15,16]. Recent meta-analyses have provided compelling evidence for the benefits of n-3 fatty acid supplementation on muscle outcomes. Huang et al. [14] conducted a comprehensive meta-analysis of randomized controlled trials and found that n-3 fatty acids supplementation resulted in significant improvements in muscle mass and strength among older people. Similarly, Cornish et al. [12] demonstrated that n-3 fatty acid supplementation, particularly when combined with resistance exercise, can increase both muscle strength and mass. Another meta-analysis by Ma et al. [17] confirmed these positive effects on muscle function with aging, while Therdyothin et al. [18] provided mechanistic insights into how n-3 fatty acids may counteract sarcopenia. These findings have been extended in randomised controlled trials, which demonstrated that n-3 fatty acid supplementation (4 g/day of fish or krill oil) can increase both muscle strength and mass [19,20]. While these results are promising, it remains unclear whether variations in n-3 fatty acids intake within the normal dietary range (1–2 g/day), affects muscle strength and mass [19,21,22].

Limited epidemiological evidence exists examining this relationship. Two epidemiological studies have identified positive correlations between oily fish consumption and grip strength in both men and women [23,24]. Another study examined the association of dietary n-3 fatty acid intake with muscle strength in 18,278 adults, finding that higher n-3 fatty acid intake was associated with lower odds of low hand grip strength. This study relied on a single 24 -h dietary recall, did not investigate muscle mass, and was not restricted to older adults [25]. Another small study of 5,529 participants (2,449 men and 3,080 women) aged 65 and older found that women, but not men, who consumed EPA and DHA at or above the adequate intake level (150 mg/d for women aged 65–74 years and 140 mg/d for women aged 75 years), also measured via a single 24 -h dietary recall, had a significantly lower likelihood of having low grip strength compared to those below the adequate intake level, with no measure of muscle mass in this study [26].

The dietary intake ratio of n-6 fatty acid to n-3 fatty acid (n-6/n-3 fatty acid intake ratio) may also be critical for muscle health, as both fatty acid families compete for enzymatic pathways and have opposing inflammatory effects [27]. Western diets typically have elevated n-6/n-3 fatty acid intake ratios (15:1 to 20:1) compared to the recommended 4:1 ratio, promoting a more pro-inflammatory state that may accelerate muscle protein breakdown and inhibit muscle protein synthesis, promoting muscle loss [27]. While both n-3 and n-6 fatty acid intake and their ratio may influence muscle during ageing, the optimal intake ratio remains unclear [28]. A recent cross-sectional study suggested an n-6/n-3 fatty acid intake ratio of 6.8 was optimal for preventing sarcopenia in women, but no cut off value was identified in men [28]. Further research supporting this sex difference, reporting stronger associations in women [29,30].

Given the uncertainty in the existing literature, the current study aims to quantify the association of dietary n-3 fatty acid intake, and the n-6/n-3 fatty acid intake ratio, with muscle mass and strength in older adults, using data from the UK Biobank study. We hypothesized that higher intake of n-3 fatty acids, and a lower n-6/n-3 fatty acid intake ratio, would be positively associated with muscle strength and mass in older adults.

## 2 Materials and methods

### 2.1 Study design

This cross-sectional study utilised data from the UK Biobank. This study was conducted in accordance with the STROBE (Strengthening the Reporting of Observational Studies in Epidemiology) recommendations [31]. From the 502,350 participants recruited into UK Biobank a subsample of 61,381 participants were included, comprising 28,187 men and 33,194 women, in the current analyses. Participants were selected for the main analysis based on the following criteria: age 60 years or older and having complete data for the outcome, predictor, and covariate variables. The primary outcomes measured were grip strength, grip strength index, and muscle mass index. The predictor variable was n-3 fatty acid intake, and the n-6/n-3 fatty acid intake ratio, while the covariates included sociodemographic factors (age, ethnicity/race, Townsend deprivation index), multimorbidity count, total energy intake, physical activity and the month of assessment. Participants completed an electronically signed consent form and a touchscreen questionnaire at the initial assessment. Physical measurements and biological samples (such as blood, urine, and saliva) were also gathered [32,33]. Ethical approval for The UK Biobank was obtained from the Northwest Multi-Centre Research Ethics Committee (Ref: 11/NW/0382) [34].

### 2.2 Outcomes assessment

Grip strength was evaluated using a Jamar J00105 hydraulic hand dynamometer, with a single measurement taken for each hand. The average grip strength from both hands was calculated in absolute terms (kg) and as a grip strength index, expressed as (kg) per meter height squared, for subsequent analyses. Muscle mass index was measured by the (Tanita) body composition analyser (Tanita BC418MA, Tokyo, Japan) using bioimpedance, the equations of Janssen et al., were used and muscle mass index, relative to height squared, calculated as kg/m^2^ [5].

For sensitivity analysis in people with sarcopenia, this was classified in accordance with the revised guidelines of the European Working Group on Sarcopenia in Older People 2 (EWGSOP2), sarcopenia, with low grip strength defined as <27 kg for men and <16 kg for women. plus, low muscle mass defined as skeletal muscle mass index <7.0 kg/m² in men and <5.5 kg/m² in women [2].

### 2.3 n-3 and n-6 fatty acid intake

The average daily intake of n-3 and n-6 fatty acids, expressed in grams per day was calculated for 61,653 participants who completed multiple online dietary assessments via the Oxford WebQ, a web-based 24 -h dietary recall tool designed for large-scale population studies [34]. The number of participants who completed each recall varied: 22,535 completed one recall, 13,785 completed two recalls, 13,057 completed three recalls, 9,853 completed four recalls, and 2,151 completed five recalls. Intake values for each fatty acid were averaged across all completed recalls per participant to estimate habitual intake. The n-6/n-3 fatty acid intake ratio was calculated for each participant by dividing their estimated average daily intake of n-6 fatty acids (g/day) by their corresponding n-3 fatty acid intake (g/day). For the final analysis, 61,381 participants aged ≥60 years with complete data on exposure, outcomes, and covariates were included. As a sensitivity analysis, we repeated the analyses restricted to participants aged ≥65 years (n = 24,711) to examine the robustness of the associations using a different age cut off for definition of older adults.

### 2.4 Covariates assessment

Age was calculated at baseline based on the date of birth recorded during the initial assessment. Sex was self-reported at the baseline. Socioeconomic status, represented by area-based deprivation, was determined using the Townsend score and was derived from the residential postcode [35]. The Townsend index is a measure of material deprivation derived from four census indicators: unemployment, lack of car ownership, lack of home ownership, and overcrowded living conditions [35]. Ethnicity was self-reported and categorised into groups including White, South Asian, Black, Chinese, or mixed ethnic backgrounds. Height was assessed, with participants not wearing shoes, utilising a wall-mounted SECA 240 stadiometers. Total energy intake was assessed using a web-based 24-h recall questionnaire and expressed in kcal per day. Physical activity was self-reported via the short-form IPAQ and classified as ‘active’ (≥600 MET-min/week) or ‘inactive’ based on standard guidelines [36]. Multimorbidity was derived from self-reported long-term conditions. At baseline, participants reported whether they had been diagnosed by a doctor with any of 43 specified long-term conditions. Individuals reporting two or more of these conditions were classified as having multimorbidity (list of conditions are presented in Table S1).

### 2.5 Statistical analysis

Cohort characteristics are presented as means and standard deviations for continuous variables, and as frequencies and percentages for categorical variables. Multiple linear regression analyses were conducted to investigate the relationship between the intake of n-3 fatty acids with grip strength (absolute and as an index) and muscle mass index. The analyses were conducted in unadjusted (model 0) and adjusted models (model 1, model 2 and model 3). In model 1, we adjusted for age, deprivation index, ethnicity, assessment month, and lifestyle factors (smoking and alcohol intake). In model 2, we adjusted for all the above confounding factors plus multimorbidity. In model 3, we additionally adjusted for total energy intake and physical activity. Analysis in model 3 was replicated in a sensitivity analysis in people with sarcopenia, as defined by EWGSOP2 criteria [2]. Additionally, as the definition of older age varies, 60 or 65 years of age, we examined the association of n-3 fatty intake and the n-6/n-3 fatty acid intake ratio with muscle outcomes in people 65 years or over as a sensitivity analysis in model 3. All analyses were stratified by sex because of the differences in grip strength and muscle mass between men and women. Analysis was performed using STATA MP18 (Stata Crop LLC, College Statin, Texas) and the significance level was at p < 0.05.

## 3 Results

From the 502,350 participants recruited to the UK Biobank, this study included individuals aged 60 years or older. Of the total, 217,452 participants met the age criterion, and 211,979 had complete data available for all outcomes. The final analysis included 61,381 individuals (28,187 men and 33,194 women) who meet all inclusion criteria and had complete data for all variables. The mean intake of n-3 fatty acids in all participants was 2.13 g/day and the mean n-6/n-3 fatty acid intake ratio was 5.78. A detailed summary of the participant selection process, including exclusions, is presented in Figure S1. The cohort characteristics of the included participants categorized based on n-3 fatty acids intake are presented in Table S2. Additionally, the characteristics of participants with sarcopenia are presented in Table S3.

### 3.1 Grip Strength (kg)

#### 3.1.1 n-3 fatty acid intake

**Fig. 1:**
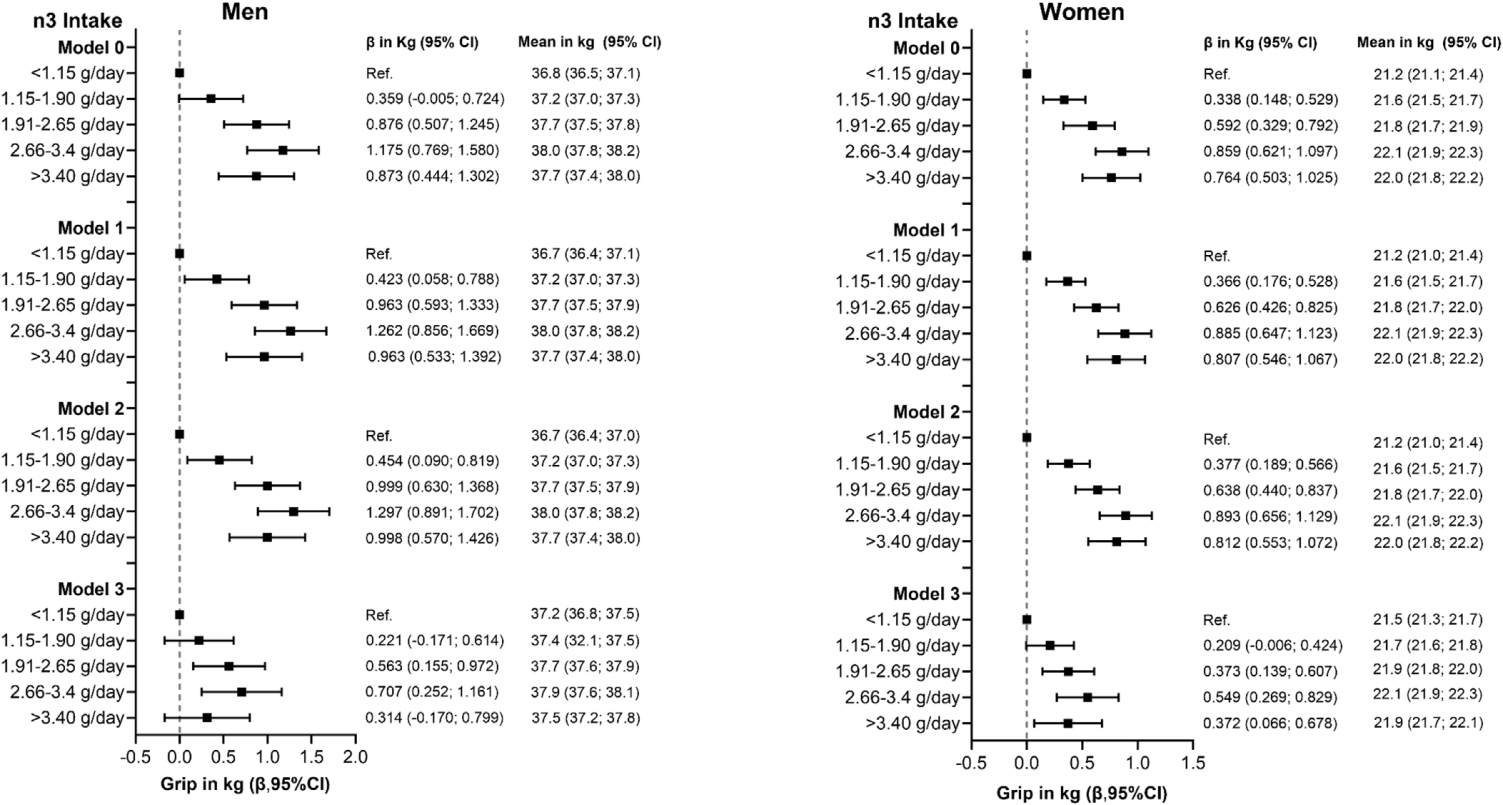
Association of n-3 fatty acid intake with handgrip strength. Data presented as beta coefficients and means with their 95% CI. The reference group was set as the lowest intake of n3 fatty acid (<1.15 g/day). Analysis was unadjusted for Model 0, and Model 1 was adjusted for age, deprivation index, ethnicity, assessment month, and lifestyle factors (smoking and alcohol intake), Model 2 additionally was adjusted for multimorbidity, Model 3 further was adjusted for energy intake and physical activity.

The associations between n-3 fatty acid intake grip and strength are shown in Table 1, Table 2 and Fig. 1. A significant positive trend was observed across all models, with grip strength increasing across quintiles of n-3 intake in both men and women. The strength of association was consistent from the unadjusted model (Model 0) to the fully adjusted model (Model 2), with beta coefficients ranging from 0.269 to 0.290 in men and 0.215 to 0.223 in women. After further adjustment for energy intake and physical activity (Model 3), the associations were attenuated but remained significant (β = 0.114 in men and 0.115 in women). In absolute terms, grip strength was 1.00 kg higher in men and 0.8 kg higher in women in the highest versus lowest intake quintile. No significant associations were observed among individuals with sarcopenia (Tables S6 and S7). When the analysis was restricted to individuals aged 65 and older, no significant associations were observed between n-3 fatty acid intake and grip strength in either men or women, regardless of sarcopenia status (Tables S14 and S15).

**Table 1:** The association of n-3 fatty acid intake with hand grip strength and muscle mass in older adult men with physical activity.

Outcomes	Model 0		Model 1		Model 2		Model 3	
Grip strength (kg)	B (95% CI)	p	B (95% CI)	p	B (95% CI)	p	B (95% CI)	p
n-3 < 1.15 g/day	Ref.							
n-3 1.15−1.90 g/day	0.359 (−0.005; 0.724)	0.053	0.423 (0.058; 0.788)	0.024	0.454 (0.090; 0.819)	0.014	0.221 (−0.171; 0.614)	0.269
n-3 1.91−2.65 g/day	0.876 (0.507; 1.245)	<0.0001	0.963 (0.593; 1.333)	<0.0001	0.999 (0.630; 1.368)	<0.0001	0.563 (0.155; 0.972)	0.007
n-3 2.66−3.4 g/day	1.175 (0.769; 1.580)	<0.0001	1.262 (0.856; 1.669)	<0.0001	1.297 (0.891; 1.702)	<0.0001	0.707 (0.252; 1.161)	0.002
n-3 > 3.40 g/day	0.873 (0.444; 1.302)	<0.0001	0.963 (0.533; 1.392)	<0.0001	0.998 (0.570; 1.426)	<0.0001	0.314 (−0.170; 0.799)	0.204
Trend for quintiles	0.269 (0.188; 0.351)	<0.0001	0.285 (0.204; 0.366)	<0.0001	0.290 (0.209; 0.371)	<0.0001	0.114 (0.018; 0.210)	0.019
Grip Strength Index (kg/m^2^)								
n-3 < 1.15 g/day	Ref.							
n-3 1.15−1.90 g/day	0.079 (−0.122; 0.281)	0.440	0.111 (−0.090; 0.313)	0.281	0.126 (−0.074; 0.328)	0.218	0.055 (−0.162; 0.272)	0.618
n-3 1.91−2.65 g/day	0.260 (0.056; 0.464)	0.012	0.303 (0.098; 0.508)	0.004	0.321 (0.117; 0.525)	<0.002	0.216 (−0.009; 0.442)	0.061
n-3 2.66−3.4 g/day	0.378 (0.154; 0.603)	0.001	0.422 (0.197; 0.647)	<0.0001	0.439 (0.214; 0.663)	<0.0001	0.288 (0.037; 0.540)	0.025
n-3 > 3.40 g/day	0.210 (−0.026; 0.447)	0.082	0.254 (0.017; 0.492)	0.036	0.272 (0.352; 0.509)	0.024	0.120 (−0.147; 0.389)	0.377
Trend for quintiles	0.081 (0.036; 0.126)	<0.0001	0.089 (0.044; 0.134)	<0.0001	0.092 (0.047; 0.136)	<0.0001	0.053 (0.000; 0.106)	0.046
Muscle Mass Index (kg/m^2^)								
n-3 < 1.15 g/day	Ref.							
n-3 1.15−1.90 g/day	0.080 (0.042; 0.117)	<0.0001	0.088 (0.050; 0.125)	<0.0001	0.084 (0.046; 0.121)	<0.0001	0.035 (−0.004; 0.075)	0.082
n-3 1.91−2.65 g/day	0.135 (0.097; 0.173)	<0.0001	0.145 (0.107; 0.183)	<0.0001	0.140 (0.103; 0.178)	<0.0001	0.037 (−0.003; 0.079)	0.073
n-3 2.66−3.4 g/day	0.174 (0.132; 0.215)	<0.0001	0.185 (0.143; 0.226)	<0.0001	0.180 (0.139; 0.221)	<0.0001	0.042 (−0.003; 0.088)	0.073
n-3 > 3.40 g/day	0.188 (0.144; 0.232)	<0.0001	0.199 (0.155; 0.243)	<0.0001	0.194 (0.151; 0.238)	<0.0001	0.050 (−0.001; 0.100)	0.043
Trend for quintiles	0.044 (0.036; 0.052)	<0.0001	0.046 (0.038; 0.054)	<0.0001	0.045 (0.037; 0.054)	<0.0001	0.007 (−0.001; 0.017)	0.115

Results are presented as beta coefficients with corresponding 95% confidence intervals (95% CI) for associations between n-3 fatty acid intake and grip strength or muscle mass. Model 0 was unadjusted. Model 1 was adjusted for age, deprivation index, ethnicity, assessment month, and lifestyle factors (smoking and alcohol intake). Model 2 included additional adjustment for multimorbidity, and Model 3 further adjusted for total energy intake and physical activity. Tests for trend across quintiles reflect the change in grip strength or muscle mass per one-quintile increase in n-3 fatty acid intake.

**Table 2:** The association between n-3 fatty acid intake and hand grip strength and muscle mass in older adult women with physical activity.

Outcomes	Model 0		Model 1		Model 2		Model 3	
Grip strength (kg)	B (95% CI)	p	B (95% CI)	p	B (95% CI)	p	B (95% CI)	p
n-3 < 1.15 g/day	Ref.						0	
n-3 1.15−1.90 g/day	0.338 (0.148; 0.529)	<0.0001	0.366 (0.176; 0.528)	0.024	0.377 (0.189; 0.566)	<0.0001	0.209 (−0.006; 0.424)	0.057
n-3 1.91−2.65 g/day	0.592 (0.329; 0.792)	<0.0001	0.626 (0.426; 0.825)	<0.0001	0.638 (0.440; 0.837)	<0.0001	0.373 (0.139; 0.607)	0.002
n-3 2.66−3.4 g/day	0.859 (0.621; 1.097)	<0.0001	0.885 (0.647; 1.123)	<0.0001	0.893 (0.656; 1.129)	<0.0001	0.549 (0.269; 0.829)	0.000
n-3 > 3.40 g/day	0.764 (0.503; 1.025)	<0.0001	0.807 (0.546; 1.067)	<0.0001	0.812 (0.553; 1.072)	<0.0001	0.372 (0.066; 0.678)	0.017
Trend for quintiles	0.215 (0.161;0.268)	<0.0001	0.222 (0.169; 0.275)	<0.0001	0.223 (0.170; 0.276)	<0.0001	0.115 (0.051; 0.180)	0.000
Grip Strength Index (kg/m^2^)								
n-3 < 1.15 g/day	Ref.							
n-3 1.15−1.90 g/day	0.144 (0.030; 0.258)	0.013	0.158 (0.044; 0.273)	0.006	0.165 (0.518; 0.278)	0.004	0.102 (−0.026; 0.232)	0.121
n-3 1.91−2.65 g/day	0.253 (0.133; 0.373)	<0.0001	0.271 (0.151; 0.391)	<0.0001	0.278 (0.159; 0.397)	<0.0001	0.191 (0.050; 0.332)	0.008
n-3 2.66−3.4 g/day	0.389 (0.246; 0.532)	<0.0001	0.403 (0.260; 0.546)	<0.0001	0.407 (0.265; 0.549)	<0.0001	0.288 (0.119; 0.456)	0.001
n-3 > 3.40 g/day	0.341 (0.184; 0.498)	<0.0001	0.363 (0.207; 0.520)	<0.0001	0.367 (0.211; 0.523)	<0.0001	0.204 (0.020; 0.388)	0.029
Trend for quintiles	0.097 (0.065; 0.129)	<0.0001	0.101 (0.069; 0.133)	<0.0001	0.101 (0.069; 0.133)	<0.0001	0.063 (0.024; 0.102)	0.001
Muscle Mass Index (kg/m^2^)								
n-3 < 1.15 g/day	Ref.							
n-3 1.15−1.90 g/day	0.072 (0.049; 0.096)	<0.0001	0.072 (0.048; 0.095)	<0.0001	0.071 (0.047; 0.094)	<0.0001	0.035 (0.009; 0.062)	0.008
n-3 1.91−2.65 g/day	0.113 (0.088; 0.137)	<0.0001	0.112 (0.087; 0.137)	<0.0001	0.111 (0.086; 0.136)	<0.0001	0.023 (−0.004; 0.052)	0.104
n-3 2.66−3.4 g/day	0.131 (0.101; 0.160)	<0.0001	0.130 (0.101; 0.160)	<0.0001	0.130 (0.100; 0.159)	<0.0001	0.008 (−0.026; 0.042)	0.640
n-3 > 3.40 g/day	0.126 (0.094; 0.158)	<0.0001	0.125 (0.093; 0.157)	<0.0001	0.125 (0.092; 0.157)	<0.0001	−0.005 (−0.043; 0.031)	0.759
Trend for quintiles	0.032 (0.025; 0.038)	<0.0001	0.031 (0.025; 0.038)	<0.0001	0.031 (0.025; 0.038)	<0.0001	−0.005 (−0.013; 0.002)	0.148

Results are presented as beta coefficients with corresponding 95% confidence intervals (95% CI) for associations between n-3 fatty acid intake and grip strength or muscle mass. Model 0 was unadjusted. Model 1 was adjusted for age, deprivation index, ethnicity, assessment month, and lifestyle factors (smoking and alcohol intake). Model 2 included additional adjustment for multimorbidity, and Model 3 further adjusted for total energy intake and physical activity. Tests for trend across quintiles reflect the change in grip strength or muscle mass per one-quintile increase in n-3 fatty acid intake.

#### 3.1.2 n-6/n-3 fatty acid intake ratio

The associations between the n-6/n-3 fatty acid intake ratio and grip strength are presented in Tables S8 and S9. No significant associations were observed between the n-6/n-3 ratio fatty acid intake ratio and grip strength in men but in women there was a significant negative association (β = –0.081 kg). No significant associations were found among individuals with sarcopenia (Tables S12 and S13). In participants aged 65 and older, the n-6/n-3 fatty acid intake ratio was not associated with grip strength in either sex, including those with sarcopenia (Tables S16 and S17).

**Fig. 2:**
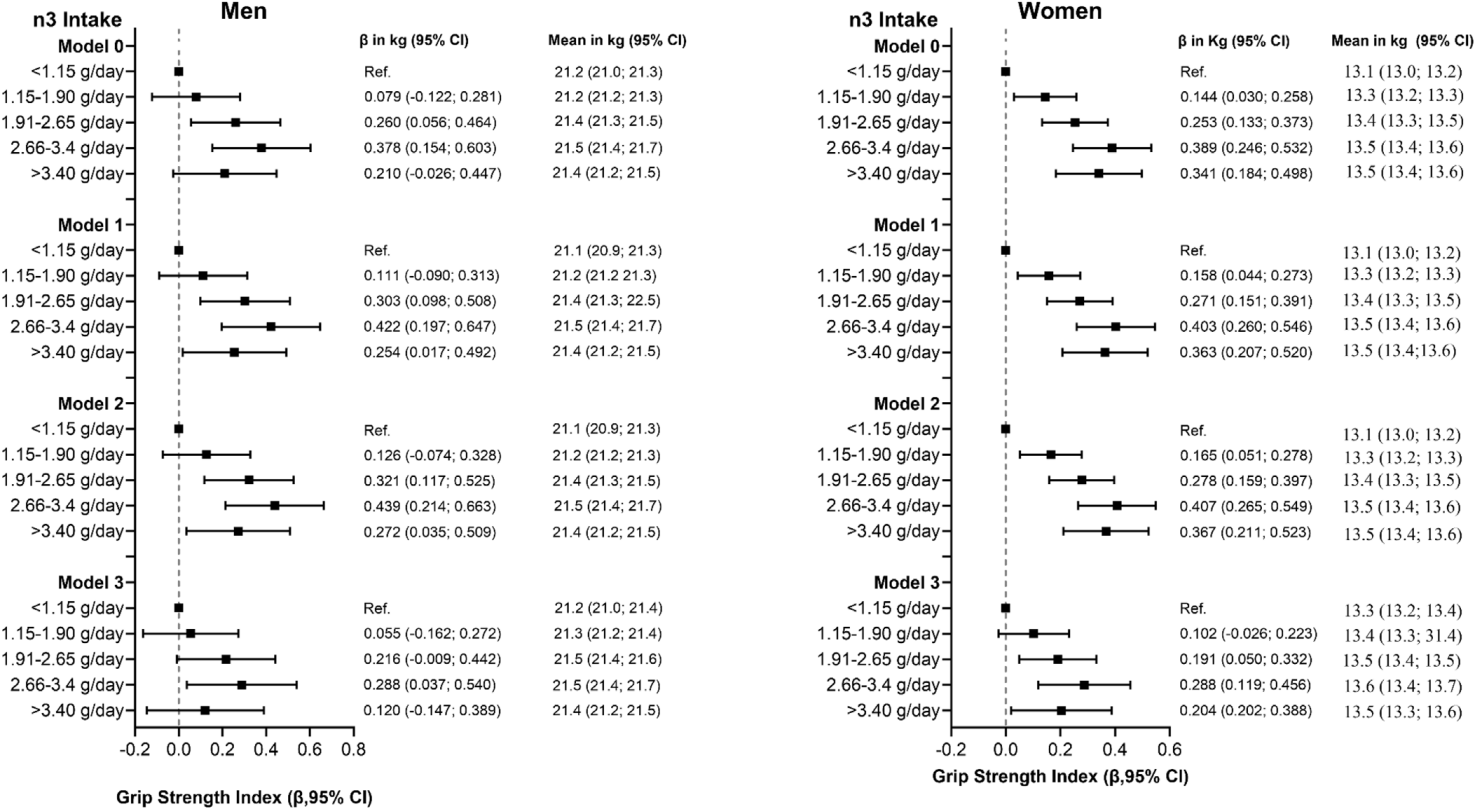
Association of n-3 fatty acid intake with handgrip strength index. Data presented as beta coefficients and means with their 95% CI. The reference group was set as the lowest intake of n3 fatty acid (<1.15 g/day). Analysis was unadjusted for Model 0, and Model 1 was adjusted for age, deprivation index, ethnicity, assessment month, and lifestyle factors (smoking and alcohol intake), Model 2 additionally was adjusted for multimorbidity, Model 3 further was adjusted for energy intake and physical activity.

### 3.2 Handgrip strength index

#### 3.2.1 n-3 fatty acid intake

Similar patterns were observed when grip strength was expressed as a height-adjusted index (kg/m²), consistent with results based on absolute grip strength (Table 1, Table 2). A significant positive trend across quintiles of n-3 fatty acid intake was found in all models, with beta coefficients for trend ranging from 0.053 to 0.092 kg/m² in men and 0.063 to 0.101 kg/m² in women (Table 1, Table 2). No associations were observed in individuals with sarcopenia (Tables S6 and S7). Among participants aged ≥65, no associations were found in either sex, including those with sarcopenia (Tables S14 and S15).

#### 3.2.2 n-6/n-3 fatty acid intake ratio

The associations between n-6/n-3 fatty acid intake ratio and grip strength index are presented in Tables S8 and S9. There were no associations between n-6/n-3 fatty acid intake ratio and grip strength index in either men or women. Similarly, in people with sarcopenia there were no associations in either men or women (Table S12 and S13). In people aged ≥ 65, there were no associations in either men or women, and similar results were observed in people with sarcopenia (Table S16 and S17).

### 3.3 Muscle mass index

#### 3.3.1 n-3 fatty acid intake

**Fig. 3:**
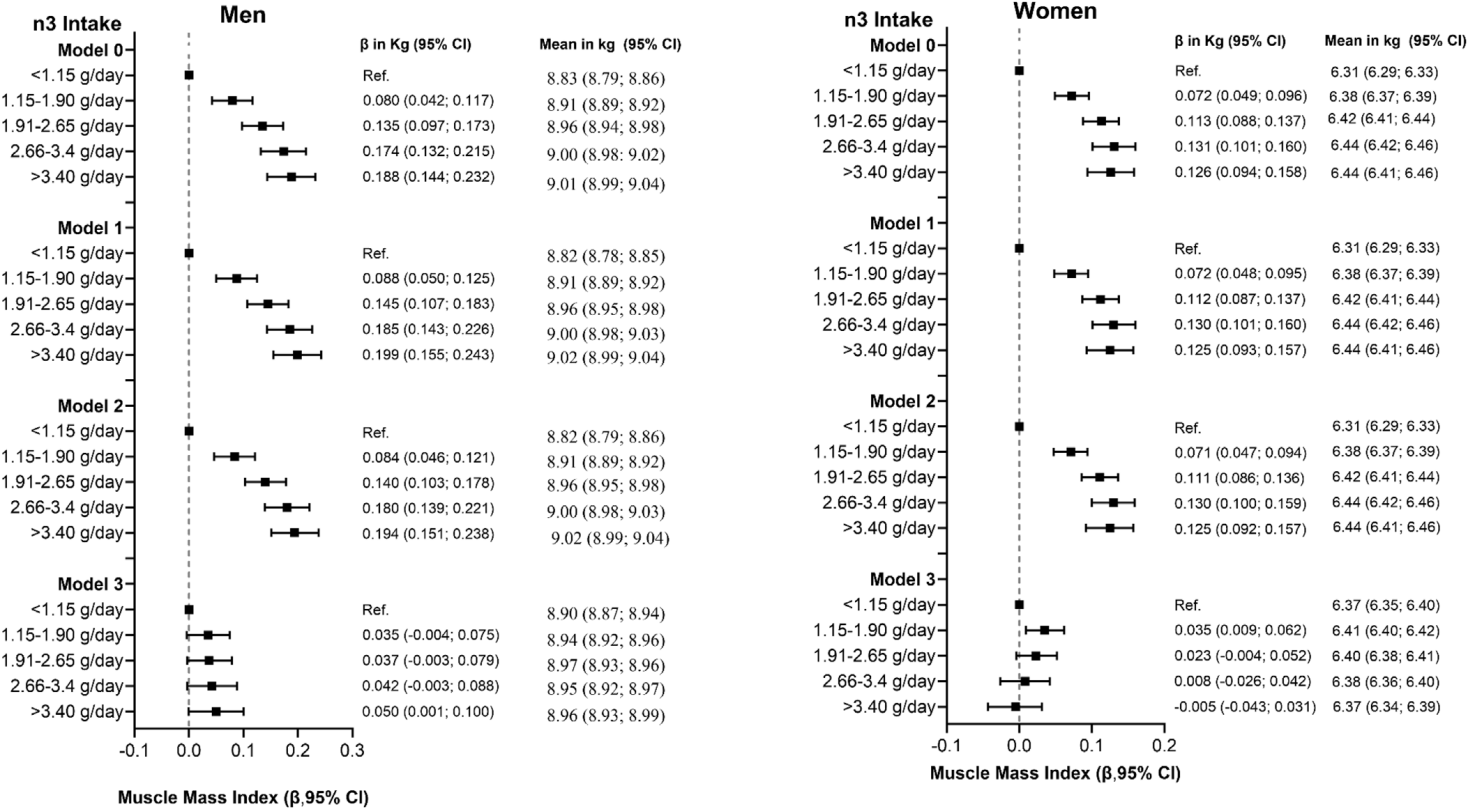
Association of n-3 fatty acid intake with muscle mass index. Data presented as beta coefficients and means with their 95% CI. The reference group was set as the lowest intake of n3 fatty acid (<1.15 g/day). Analysis was unadjusted for Model 0, and Model 1 was adjusted for age, deprivation index, ethnicity, assessment month, and lifestyle factors (smoking and alcohol intake), Model 2 additionally was adjusted for multimorbidity, Model 3 further was adjusted for energy intake and physical activity.

Results for muscle mass index are presented in Table 1, Table 2 and Fig. 3. A consistent and significant trend toward higher muscle mass index across increasing quintiles of n-3 fatty acid intake was observed from the unadjusted model (Model 0) through to Model 2 (adjusted for socio-demographic, lifestyle, and health-related covariates), with beta values ranging from 0.044 to 0.046 kg/m² in men and 0.031 to 0.032 kg/m² in women. After full adjustment, including energy intake and physical activity (Model 3), the association was fully attenuated in women (β = –0.005 kg/m²) and men (β = 0.007 kg/m²). Among people with sarcopenia, a positive association was found in men (β = 0.197 kg/m²), with no association in women (β = –0.062 kg/m²) (Tables S6 and S7). In participants aged ≥ 65, no associations were observed in either sex, including those with sarcopenia (Tables S14 and S15).

#### 3.3.2 n-6/n-3 fatty acid intake ratio

No evidence of an association was found between the n-6/n-3 fatty acid intake ratio and muscle mass index in women, but a negative association was seen in men (β = -0.016) (Tables S8 and S9). Similarly, no associations were observed among individuals with sarcopenia, either men or women, (Tables S12 and S13), or among participants aged ≥65 (Tables S16 and S17).

## 4 Discussion

This study examined the relationship between n-3 fatty acid intake, and the n-6/n-3 fatty acid intake ratio, and measures of muscle strength and mass in a large sample of 61,381 individuals aged 60 years and older, including a subgroup aged ≥65 years. The findings provide valuable insights into the role of both n-3 fatty acid intake and the n-6/n-3 fatty acid intake ratio in muscle health among older adults. A positive association was observed between n-3 fatty acid intake and handgrip strength in both men and women, even after adjusting for key confounders such as socio-demographic, lifestyle, and health-related factors. However, these associations were not present among individuals with sarcopenia. Regarding muscle mass index, n-3 fatty acid intake was not associated with muscle mass in men, or in women, in fully adjusted models, although among those with sarcopenia, a positive association was observed in men. No consitent associations were found between the n-6/n-3 fatty acid intake ratio and muscle mass index or strength, although negative associations were seen with muscle mass index in men and absolute grip strength in women.

There are a few other studies which have examined the relationship between n-3 fatty acid or oily fish intake and muscle strength, with which we can compare our findings. The current data align with findings of Robinson et al., who analyzed 2,983 older adults from Hertfordshire, UK and found that each additional portion of fatty fish consumed per week was associated with a higher grip strength of 0.43 kg in men (95% CI: 0.13−0.74) and 0.48 kg in women (95% CI: 0.24−0.72), after adjusting for height, age, and birth weight [23]. Similar results have been found in analysis of the UK Biobank data, where grip strength in both sexes was higher with each additional portion of oily fish consumed per week (p = 0.0001) [24]. While these comparisons are useful, oily fish contains nutrients such as protein and vitamin D and so these data do not isolate the potential role of n-3 fatty acids per se. In contrast, other work in Korea, in people aged 65+, investigated the relationship between the intake of n-3 fatty acids (EPA and DHA) and grip strength finding that in older women, but not men, consuming EPA and DHA at or above an adequate intake had significantly lower odds of low grip strength (OR = 0.777, 95% CI: 0.616−0.979, p = 0.0322), after adjusting for factors like BMI, household income, and smoking status [25]. In contrast, the current study observed a consistent relationship between n-3 fatty acid intake and grip strength in both men and women. Further work in Korea focused on a broader adult population (aged 19+) and included 18,278 participants, finding that higher n-3 fatty acid intake was associated with a reduced odds of low grip strength in both men and women. For men, the odds ratio was 1.42 (95% CI: 1.17–1.72), and for women, it was 1.61 (95% CI: 1.37–1.89), comparing the highest to the lowest quartile of n-3 fatty acid intake [26]. With the addition of the current data it appears, therefore, that a higher n-3 fatty acid intake is associated with a higher muscle strength in both men and women. The current study, extends these findings by demonstrating that there was no clear relationship between n-3 fatty acid intake and grip strength in people with sarcopenia or in those over 65 years of age. This lack of association in those over 65 years and/or those with sarcopenia suggests n-3 fatty acids may help prevent, but not reverse, muscle decline although the magnitude of prevention is likely to be small within the current intake ranges and requires confirmation in appropriately designed trials with no studies currently in people with sarcopenia.

Indeed the current study and other works suffer the limitation that they are cross sectional in design and so causality cannot be determined, but the associations in the general older adult population are supported by previous RCTs, using supplementary n-3 fatty acids, in this area. Indeed, a randomized controlled trial, in older adults, showed a 2.3 kg (95% CI: 0.8–3.7 kg; P < 0.01) increase in handgrip strength after six months of fish oil supplementation [19]. Similarly, a study reported a 10.9% (95% CI: 8.3–13.6%) increase in grip strength in older adults after six months of krill oil supplementation [20]. A meta-analysis further supported these findings, showing significant improvements in grip strength with n-3 fatty acid supplementation among healthy individuals [17]. There are no RCTs, to our knowledge, of n-3 fatty acid treatment in people with sarcopenia a research gap that needs to be filled. These aforementioned RCTs all used a daily dose of n-3 fatty acid of around 4 g/day, which is far in excess of the dietary intakes of n-3 fatty acid observed in the current study and is also primarily EPA and DHA rather than other n-3 fatty acids. This likely explains the lower effect size seen in the current study cross-sectional data compared to the data from RCTs. Indeed the minimal clinically important difference (MCID) for grip strength has been estimated to be around 5.0–6.5 kg [37]. This suggests that the direct impact of changes in n-3 fatty acid intake, within the normally dietary range, on muscle function is limited, though even small gains may still be relevant for overall health and aging.

In contrast to the data on grip strength, the evidence for an effect of n-3 fatty acid on muscle mass is more ambiguous. In our study no association was observed between n-3 fatty acid intake and muscle mass index in men or in women. This disagrees with findings from previous research using more robust methodologies to quantify muscle mass, which reported that n-3 fatty acid may improve muscle mass [18]. For example, a meta-analysis found n-3 fatty acid supplementation positively influenced muscle mass and walking speed in older adults, particularly at doses exceeding 2 g/day [14]. Another study reported a 3.6% increase in thigh muscle volume, measured using MRI, after 6 months of fish oil supplementation in older adults [19]. Another study, despite finding no significant effect of 6 months krill oil supplementation in older adults on muscle mass, measured using bioelectrical impedance analysis (BIA), observed an increase in vastus lateralis muscle thickness, measured via ultrasound measurements [20]. Much of the ambiguity in our findings and the wider literature may be as muscle mass is estimated by BIA. While BIA is commonly used for body composition assessment, it has limitations, including sensitivity to hydration status and potential accuracy issues, which should be considered when interpreting the results [3]. In sensitivity analyses among adults aged ≥65 years, no associations were observed between n-3 fatty acid intake and muscle mass in either sex, including individuals with sarcopenia, although as mentioned previously participant numbers were low in these analyses. A positive association was, however, noted in men, but not women, aged over 60 years with sarcopenia, tentatively indicating a potential therapeutic effect in this population. This is worthy of follow up in subsequent appropriately designed trials.

Previous research has indicated the importance of the n-6/n-3 fatty acid intake ratio in chronic disease prevention [27], but its role in sarcopenia is less well explored. In the current study, we found no consistent associations between the n-6/n-3 fatty acid intake ratio and muscle strength or muscle mass in either men or women, although relatively weak negative associations were seen with muscle mass index in men and with grip strength in women. No other associations were observed across both age groups (≥60 and ≥65 years), including individuals with sarcopenia. These findings are broadly consistent with prior studies that also reported no significant associations between the n-6/n-3 fatty acid intake ratio and muscle health or sarcopenia status [8,10,38,39]. Overall, our results contribute to the growing body of evidence suggesting that the n-6/n-3 fatty acid intake ratio may not have a meaningful impact on muscle health in older adults, regardless of sex, although the associations that were observed may be worthy of further follow up.

The mechanisms by which n-3 fatty acids might influence muscle strength, but not necessarily muscle mass, are multifaceted. For example, n-3 fatty acid, particularly DHA, play a crucial role in supporting neural function by maintaining membrane phospholipid integrity, which is essential for receptor activity and signal transduction [25,40]. Improved muscle power and excitability through increased electromyography and M-wave, a measure of excitability, measurements have been observed in younger adults following n-3 fatty acid supplementation [41,42]. Furthermore, after 6 months krill oil supplementation in older adults M-wave was increased by 17.4% in the krill oil group compared to the control group [20]. There is also evidence that n-3 fatty acid may contribute to improved mitochondrial function, enhanced blood flow, and anti-inflammatory effects, all of which could support muscle strength [43]. On top of this, n-3 fatty acids may help prevent loss of muscle strength due to their anti-inflammatory effects while n-6 fatty acids can potentially promote muscle loss by triggering inflammation. Indeed, metabolites of n-6 fatty acids are linked to increased levels of pro-inflammatory markers such as interleukin-1 (IL-1), interleukin-6 (IL-6), tumor necrosis factor (TNF), and C-reactive protein (CRP) [10,44,45]. These markers tend to rise with higher n-6 fatty acid intake and decrease with greater n-3 fatty acid intake, highlighting the potential importance of the balance in n-6 and n-3 fatty acid intake. In spite of these differing inflammatory effects, these are not reflected in consistent associations of the n-6/n-3 fatty acid intake ratio with muscle strength or mass in the current data.

It is prudent to consider the strengths and limitations of the current study. The large sample size enhances the statistical power of the findings and the inclusion of both men and women provides a comprehensive understanding of sex-specific effects, and the adjustment for various confounding factors strengthens the internal validity of the study. Despite its strengths, the study has notable limitations. Our subgroup analysis in people with sarcopenia and those over 65 years of age must be treated with caution as the participants numbers were, however, low. The use of self-reported dietary data for n-3 and n-6 fatty acid intake may introduce measurement errors, affecting the accuracy of the observed associations [33], and does not allow us to understand the contribution of specific n-3 and n-6 fatty acids. Additionally, the cross-sectional nature of the study design prohibits the establishment of causation and the potential for residual confounding and reverse causation cannot be entirely ruled out. While the study benefits from the large cohort of the UK Biobank, caution is needed when generalizing these findings to broader populations. Although we included adults aged 60 and above, a sensitivity analysis restricted to those aged ≥65 showed no major differences in findings, reinforcing the robustness of the results, although in our sensitivity analysis participant numbers were often low. Finally, the characteristics of UK Biobank participants, who are typically healthier and less diverse than the general population, limit the study's external validity [46]. However, this demographic skew is unlikely to significantly affect the relationships between the variables being studied, meaning the observed associations within the cohort remain robust. The current analysis, although performing stratified analysis by sex, did not formally test for sex differences which may be present. There remains the possibility that current population-based recommendations for n-3 fatty acid intake may not account for sex-specific physiological differences, suggesting that sex-specific recommendations for n-3 fatty acid intake may need further investigation.

In summary, the current data demonstrated a relationship between n-3 fatty acid intake and higher grip strength, although the magnitude of association was small, with no clear pattern of associations found with n-6/n-3 fatty acid intake ratio. This suggests that within the typical dietary range, n-3 fatty acid consumption may not offer significant public health benefits for improving muscle strength, and higher dose supplementation strategies with EPA and DHA specifically may be needed to realise the benefits of increased n-3 fatty acid intake.

## CRediT authorship contribution statement

**Maha Y Timraz:** Writing – original draft, Visualization, Validation, Software, Resources, Methodology, Investigation, Funding acquisition, Formal analysis, Data curation, Conceptualization, Writing – review & editing. **Marion T. Guerrero:** Formal analysis, Software, Visualization. **Terry J Quinn:** Supervision, Writing – review & editing. **Emilie Combet:** Supervision, Writing – review & editing. **Carlos Celis-Morales:** Supervision, Software, Resources, Methodology, Conceptualization, Writing – review & editing. **Stuart R. Gray:** Visualization, Validation, Supervision, Project administration, Methodology, Conceptualization, Writing – review & editing.

## Ethical approval

The UK Biobank study received approval from the North West Multi-Centre Research Ethics Committee, and all participants provided written informed consent to take part in the research. The study protocol is accessible online at (http://www.ukbiobank.ac.uk/). This research was conducted under UK Biobank application number 71392.

## Declaration of Generative AI and AI-assisted technologies in the writing process

During the preparation of this work, the author (s) used ChatGPT for paraphrasing, improving clarity, and refining the structure of the manuscript. After using this tool, the author(s) reviewed and edited the content as needed and take(s) full responsibility for the content of the publication.

## Funding

The UK Biobank has been supported by several organisations, including the 10.13039/100010269Wellcome Trust, 10.13039/501100007155Medical Research Council, 10.13039/501100003921Department of Health, 10.13039/100012095Scottish Government, 10.13039/501100004186Northwest Regional Development Agency, Welsh Assembly Government, and the 10.13039/501100000274British Heart Foundation. All authors retained full responsibility for the decision to submit this work for publication. M-T acknowledges financial support for her PhD studies from the Government of Saudi Arabia.

## Declaration of competing interest

None.
