# Supplementary material for: The association of n-3 fatty acid intake with muscle mass and strength in older adults: A cross-sectional analysis of the UK biobank data
Source: J Nutr Health Aging. 2025 Jun 27;29(9):100622. doi: 10.1016/j.jnha.2025.100622 (PMC12695521; doi:10.1016/j.jnha.2025.100622)
Supplement: Supplementary file 1 [file mmc1.docx]

**Supplementary materials:**

**Table S1: Overview of Self-Reported Long-Term Health Conditions Included in the Multimorbidity Count**

| **Long term condition grouping** | **Conditions included as reported by participants** |
| --- | --- |
| 1. Painful conditions | Back pain  Joint pain  Back pain  Joint pain  Headaches (not migraine)  Sciatica  Plantar fasciitis  Carpal tunnel syndrome  Fibromyalgia  Arthritis  Shingles  Disc problem  Prolapsed disc/slipped disc  Spine arthritis/spondylitis  Ankylosing spondylitis  Back problem  Osteoarthritis  Gout  Cervical spondylosis  Trigeminal neuralgia  Disc degeneration  Trapped nerve/compressed nerve |
| 1. Hypertension | Hypertension  Essential Hypertension |
| 1. Depression | Depression  Postnatal Depression |
| 1. Asthma | Asthma |
| 1. Atrial Fibrillation | Atrial Fibrillation |
| 1. Coronary Heart Disease | Heart attack/Myocardial Infarction  Angina |
| 1. Dyspepsia | Gastro-oesophageal reflux (GORD)/gastric reflux  Oesophagitis /Barrett's oesophagus  Gastric stomach ulcers  Gastric erosions/gastritis  Duodenal ulcer  Dyspepsia/indigestion  Hiatus hernia  Helicobacter pylori |
| 1. Diabetes | Diabetic nephropathy  Diabetic neuropathy/ulcers  Diabetes  Type 1 diabetes  Type 2 diabetes  Diabetic eye disease |
| \| 1. Thyroid disorders \| \| --- \| | Thyroid problem (not cancer)  Hyperthyroidism/thyrotoxicosis  Hypothyroidism/myxoedema  Grave’s disease  Thyroid goitre  Thyroiditis |
| 1. Connective tissue disorders | Myositis/myopathy  Systemic Lupus Erythematosus  Connective tissue disorder  Sjogrens syndrome/sicca syndrome  Dermatopolymyositis  Scleroderma/systemic sclerosis  Rheumatoid arthritis  Psoriatic arthropathy  Dermatomyositis  Polymyositis  Polymyalgia Rheumatica  Malabsorption/coeliac disease |
| 1. Chronic Obstructive Pulmonary Disease (COPD) | COPD/chronic obstructive airways disease  Emphysema/ chronic bronchitis  Emphysema |
| 1. Anxiety | Anxiety/panic attacks  Nervous breakdown  Post-traumatic stress disorder  Obsessive compulsive disorder  Stress  Insomnia  Psychological/psychiatric problem |
| 1. Irritable bowel syndrome | Irritable bowel syndrome |
| 1. Alcohol problems | Alcohol dependency  Alcoholic liver disease/alcoholic cirrhosis |
| 1. Other psychoactive substance abuse | Opioid dependency  Other substance abuse/dependency |
| 1. Treated constipation | Constipation |
| 1. Stroke/Transient Ischaemic Attack (TIA) | Stroke  TIA  Subarachnoid haemorrhage  Brain haemorrhage  Ischaemic stroke |
| 1. Chronic kidney disease | Polycystic kidney  Diabetic nephropathy  Renal/kidney failure  Renal failure requiring dialysis.  Renal failure not requiring dialysis.  Kidney nephropathy  Immunoglobulin A (IgA) nephropathy |
| 1. Diverticular disease | Diverticular disease  Diverticulitis |
| 1. Peripheral vascular disease | Peripheral vascular disease  Leg claudication/intermittent claudication |
| 1. Heart failure | Cardiomyopathy  Hypertrophic cardiomyopathy  Heart failure/pulmonary oedema |
| 1. Prostate disorders | Prostate problem (not cancer)  Enlarged prostate.  Benign prostatic hypertrophy |
| 1. Glaucoma | Glaucoma |
| 1. Epilepsy | Epilepsy |
| 1. Dementia | Dementia  Alzheimer’s disease  Cognitive impairment |
| 1. Schizophrenia/bipolar disorder | Schizophrenia  Mania/  Bipolar disorder  Manic depression |
| 1. Psoriasis/eczema | Eczema  Dermatitis  Psoriasis |
| 1. Inflammatory Bowel Disease | Inflammatory Bowel Disease  Crohn’s disease  Ulcerative colitis |
| 1. Migraine | Migraine |
| 1. Chronic sinusitis | Chronic sinusitis |
| 1. Anorexia or bulimia | Anorexia  Bulimia  Other eating disorders |
| 1. Bronchiectasis | Bronchiectasis |
| 1. Parkinson’s disease | Parkinson’s disease |
| 1. Multiple Sclerosis | Multiple Sclerosis |
| 1. Viral Hepatitis | Infective/viral hepatitis  Hepatitis B  Hepatitis C  Hepatitis D  Hepatitis E |
| 1. Chronic Liver disease | Oesophageal varices  Non infective hepatitis  Liver failure/cirrhosis  Primary biliary cirrhosis |
| 1. Osteoporosis | Osteoporosis |
| 1. Chronic fatigue syndrome | Chronic fatigue syndrome |
| 1. Endometriosis | Endometriosis |
| 1. Meniere’s disease | Meniere’s disease |
| 1. Pernicious Anaemia | Pernicious Anaemia |
| 1. Polycystic ovary | Polycystic ovary |
| 1. Cancer | Lifetime diagnosis |

|  | **n-3 fatty acids intake categories in g per day** | | | | | |
| --- | --- | --- | --- | --- | --- | --- |
|  | <1.15 | 1.15-1.90 | 1.91-2.65 | 2.66-3.4 | >3.40 | Total |
| **N (%)** | 6,330 (10.3%) | 23,056 (37.6%) | 17,939 (29.2%) | 8,128 (13.2%) | 5,928 (9.7%) | 61,381 (100.0%) |
| **Age (years)** | 63.9 (2.7) | 63.8 (2.7) | 63.8 (2.7) | 63.9 (2.8) | 63.9 (2.8) | 63.8 (2.7) |
| **Sex, n (%)** |  |  |  |  |  |  |
| Women | 4,282 (67.6%) | 13,004 (56.4%) | 9,122 (50.9%) | 3,942 (48.5%) | 2,844 (48.0%) | 33,194 (54.1%) |
| Men | 2,048 (32.4%) | 10,052 (43.6%) | 8,817 (49.1%) | 4,186 (51.5%) | 3,084 (52.0%) | 28,187 (45.9%) |
| **Deprivation index, n (%)** |  |  |  |  |  |  |
| Least deprived | 2,409 (38.1%) | 9,161 (39.7%) | 6,867 (38.3%) | 3,136 (38.6%) | 2,335 (39.4%) | 23,908 (39.0%) |
| Middle deprived | 2,238 (35.4%) | 8,216 (35.6%) | 6,465 (36.0%) | 2,881 (35.4%) | 2,091 (35.3%) | 21,891 (35.7%) |
| Most deprived | 1,683 (26.6%) | 5,679 (24.6%) | 4,607 (25.7%) | 2,111 (26.0%) | 1,502 (25.3%) | 15,582 (25.4%) |
| **Ethnicity, n (%)** |  |  |  |  |  |  |
| White | 6,181 (97.6%) | 22,638 (98.2%) | 17,651 (98.4%) | 7,994 (98.4%) | 5,795 (97.8%) | 60,259 (98.2%) |
| Mixed | 46 (0.7%) | 146 (0.6%) | 102 (0.6%) | 47 (0.6%) | 47 (0.8%) | 388 (0.6%) |
| South Asian | 64 (1.0%) | 174 (0.8%) | 112 (0.6%) | 47 (0.6%) | 46 (0.8%) | 443 (0.7%) |
| Black | 26 (0.4%) | 66 (0.3%) | 52 (0.3%) | 26 (0.3%) | 33 (0.6%) | 203 (0.3%) |
| Chinese | 13 (0.2%) | 32 (0.1%) | 22 (0.1%) | 14 (0.2%) | 7 (0.1%) | 88 (0.1%) |
| **Ipaq_totalpaactive** |  |  |  |  |  |  |
| Inactive | 774 (15.2%) | 2,818 (14.7%) | 2,277 (15.1%) | 1,004 (14.6%) | 678 (13.4%) | 7,551 (14.7%) |
| active | 4,327 (84.8%) | 16,373 (85.3%) | 12,784 (84.9%) | 5,891 (85.4%) | 4,371(86.6%) | 43,746 (85.3%) |
| **Smoking status, n (%)** |  |  |  |  |  |  |
| Never | 3,222 (50.9%) | 11,988 (52.0%) | 9,321 (52.0%) | 4,263 (52.4%) | 3,113 (52.5%) | 31,907 (52.0%) |
| Previous | 2,690 (42.5%) | 9,778 (42.4%) | 7,643 (42.6%) | 3,393 (41.7%) | 2,526 (42.6%) | 26,030 (42.4%) |
| Current | 418 (6.6%) | 1,290 (5.6%) | 975 (5.4%) | 472 (5.8%) | 289 (4.9%) | 3,444 (5.6%) |
| **Alcohol consumption (Units/week)** | 16.61 (19.03) | 15.96 (17.05) | 15.60 (16.41) | 16.09 (16.69) | 15.97 (17.02) | 15.94 (17.03) |
| **Multimorbidity, n (%)** |  |  |  |  |  |  |
| 0 LTC | 1,831 (28.9%) | 6,434 (27.9%) | 4,987 (27.8%) | 2,261 (27.8%) | 1,659(28.0%) | 17,172 (28.0%) |
| 1LTC | 2,145 (33.9%) | 7,909 (34.3%) | 6,145 (34.3%) | 2,808 (34.5%) | 2,032(34.3%) | 21,039 (34.3%) |
| 2+ LTCs | 2,354 (37.2%) | 8,713 (37.8%) | 6,807 (37.9%) | 3,059 (37.6%) | 2,237(37.7%) | 23,170 (37.7%) |
| **Total Energy intake (Kcal/day)** | 1,829 (317) | 2,057 (360) | 2,276(422) | 2,416 (484) | 2,489 (527) | 2,187 (454) |

**Table S2. Cohort characteristics by quintiles of n-3 fatty acid intake**

Data presented as mean and (SD) for continuous variables and as frequency observations and % for categorical variables. LTC: Long Term Condit

**Table S3: Cohort characteristics by quintiles of n-3 fatty acid intake in people with sarcopenia.**

|  | **n-3 fatty acids intake categories in g per day** | | | | | |
| --- | --- | --- | --- | --- | --- | --- |
|  | <1.15 | 1.15-1.90 | 1.91-2.65 | 2.66-3.4 | >3.40 | Total |
| **N (%)** | 183 (13.4%) | 537 (39.3%) | 382 (28.0%) | 175 (12.8%) | 89 (6.5%) | 1,366 (100.0%) |
| **Age (years)** | 64.6 (2.8) | 64.5 (2.8) | 64.4 (2.8) | 64.3 (2.8) | 64.6 (3.1) | 64.5 (2.8) |
| **Sex, n (%)** |  |  |  |  |  |  |
| Women | 158 (86.3%) | 420 (78.2%) | 276 (72.3%) | 130 (74.3%) | 69 (77.5%) | 1,053 (77.1%) |
| Men | 25 (13.7%) | 117 (21.8%) | 106 (27.7%) | 45 (25.7%) | 20 (22.5%) | 313 (22.9%) |
| **Deprivation index, n (%)** |  |  |  |  |  |  |
| Least deprived | 44 (24.0%) | 168 (31.3%) | 114 (29.8%) | 47 (26.9%) | 35 (39.3%) | 408 (29.9%) |
| Middle deprived | 67 (36.6%) | 188 (35.0%) | 125 (32.7%) | 63 (36.0%) | 27 (30.3%) | 470 (34.4%) |
| Most deprived | 72 (39.3%) | 181 (33.7%) | 143 (37.4%) | 65 (37.1%) | 27 (30.3%) | 488 (35.7%) |
| **Ethnicity** |  |  |  |  |  |  |
| White | 176 (96.2%) | 513 (95.5%) | 357 (93.5%) | 162 (92.6%) | 86 (96.6%) | 1,294 (94.7%) |
| Mixed | 3 (1.6%) | 5 (0.9%) | 9 (2.4%) | 5 (2.9%) | 0 (0.0%) | 22 (1.6%) |
| South Asian | 3 (1.6%) | 15 (2.8%) | 13 (3.4%) | 4 (2.3%) | 2 (2.2%) | 37 (2.7%) |
| Black | 0 (0.0%) | 3 (0.6%) | 2 (0.5%) | 2 (1.1%) | 1 (1.1%) | 8 (0.6%) |
| Chinese | 1 (0.5%) | 1 (0.2%) | 1 (0.3%) | 2 (1.1%) | 0 (0.0%) | 5 (0.4%) |
| **ipaq_totalpaactive** |  |  |  |  |  |  |
| Inactive | 43 (30.9%) | 128 (32.0%) | 95 (33.2%) | 41 (29.3%) | 23 (34.3%) | 330 (32.0%) |
| active | 96 (69.1%) | 272 (68.0%) | 191 (66.8%) | 99 (70.7%) | 44 (65.7%) | 702 (68.0%) |
| **Smoking status, n (%)** |  |  |  |  |  |  |
| Never | 92 (50.3%) | 296 (55.1%) | 200 (52.4%) | 79 (45.1%) | 44 (49.4%) | 711 (52.0%) |
| Previous | 75 (41.0%) | 204 (38.0%) | 163 (42.7%) | 80 (45.7%) | 38 (42.7%) | 560 (41.0%) |
| Current | 16 (8.7%) | 37 (6.9%) | 19 (5.0%) | 16 (9.1%) | 7 (7.9%) | 95 (7.0%) |
| **Alcohol consumption (Units/week)** | 11.3 (1.8) | 9.9 (1.0) | 10.3 (1.8) | 9.7 (1.5) | 10.1 (1.4) | 10.2 (1.2) |
| **Multimorbidity, n (%)** |  |  |  |  |  |  |
| 0 LTC | 23 (12.6%) | 52 (9.7%) | 42 (11.0%) | 24 (13.7%) | 9 (10.1%) | 150 (11.0%) |
| 1LTC | 48 (26.2%) | 108 (20.1%) | 86 (22.5%) | 45 (25.7%) | 28 (31.5%) | 315 (23.1%) |
| 2+ LTCs | 112 (61.2%) | 377 (70.2%) | 254 (66.5%) | 106 (60.6%) | 52 (58.4%) | 901 (66.0%) |
| **Total Energy intake (Kcal/day)** | 1,745.27 (316.8) | 1,971.46 (361.8) | 2,187.88 (440.5) | 2,361.51 (481.8) | 2,411.27 (541.2) | 2,080.31 (455.7) |

Data presented as mean and (SD) for continuous variables and as frequency observations and % for categorical variables.

UK BIOBANK Total data available

**n = 502,350**

People> 60 years old

**n = 217,452**

Available data for outcomes

(**Grip strength**) **n= 216,444**

**(Grip Index) n= 215,905**

**(Muscle Mass) n= 212,285**

**n = 216,444**

**Excluded**

Missing data for outcomes

**n = 5473**

People with data for all outcomes

**n= 211,179**

**n = 264**

Missing diet data

**n = 128,009**

Missing data for covariate

**n=22,589**

**n = 264**

Diet and outcome data available

**n= 83,970**

**n = 264**

Data available final included

**n = 61,381**

**Included**

**Figure S1 – flowchart for participants included in the study.**

**Table S4: The association of n-3 fatty acid intake with hand grip strength and muscle mass in older men.**

|  | **Model 0** | **Model 1** | **Model 2** | **Model 3** |
| --- | --- | --- | --- | --- |
| **Grip strength (kg)** | **Mean (95% CI)** | **Mean (95% CI)** | **Mean (95% CI)** | **Mean (95% CI)** |
| n-3 <1.15 g/day | 36.8 (36.5; 37.1) | 36.7 (36.4; 37.1) | 36.7 (36.4; 37.0) | 37.2 (36.8; 37.5) |
| n-3 1.15-1.90 g/day | 37.2 (37.0; 37.3) | 37.2 (37.0; 37.3) | 37.2 (37.0; 37.3) | 37.4 (37.2; 37.5) |
| n-3 1.91-2.65 g/day | 37.7 (37.5; 37.8) | 37.7 (37.5; 37.9) | 37.7 (37.5; 37.9) | 37.7 (37.6; 37.9) |
| n-3 2.66-3.4 g/day | 38.0 (37.8; 38.2) | 38.0 (37.8; 38.2) | 38.0 (37.8; 38.2) | 37.9 (37.6; 38.1) |
| n-3 >3.40 g/day | 37.7 (37.4; 38.0) | 37.7 (37.4; 38.0) | 37.7 (37.4; 38.0) | 37.5 (37.2; 37.8) |
| **Grip Strength Index (kg/m^2^)** |  |  |  |  |
| n-3 <1.15 g/day | 21.2 (21.0; 21.3) | 21.1 (20.9; 21.3) | 21.1 (20.9; 21.3) | 21.2 (21.0; 21.4) |
| n-3 1.15-1.90 g/day | 21.2 (21.2; 21.3) | 21.2 (21.2; 21.3) | 21.2 (21.2; 21.3) | 21.3 (21.2; 21.4) |
| n-3 1.91-2.65 g/day | 21.4 (21.3: 21.5) | 21.4 (21.3; 21.5) | 21.4 (21.3; 21.5) | 21.5 (21.4; 21.6) |
| n-3 2.66-3.4 g/day | 21.5 (21.4; 21.7) | 21.5 (21.4; 21.7) | 21.5 (21.4; 21.7) | 21.5 (21.4; 21.7) |
| n-3 >3.40 g/day | 21.4 (21.2: 21.5) | 21.4 (21.2; 21.5) | 21.4 (21.2.;21.5) | 21.4 (21.2; 21.5) |
| **Muscle Mass Index (kg/m^2^)** |  |  |  |  |
| n-3 <1.15 g/day | 8.83 (8.79; 8.86) | 8.82 (8.78; 8.85) | 8.82 (8.79; 8.86) | 8.90 (8.87; 8.94) |
| n-3 1.15-1.90 g/day | 8.91 (8.89; 8.92) | 8.91 (8.89; 8.92) | 8.91 (8.89; 8.92) | 8.94 (8.92; 8.96) |
| n-3 1.91-2.65 g/day | 8.96 (8.94; 8.98) | 8.96 (8.95; 8.98) | 8.96 (8.95; 8.98) | 8.94 (8.93; 8.96) |
| n-3 2.66-3.4 g/day | 9.00 (8.98: 9.02) | 9.00 (8.98; 9.03) | 9.00 (8.98; 9.03) | 8.95 (8.92; 8.97) |
| n-3 >3.40 g/day | 9.01 (8.99; 9.04) | 9.02 (8.99; 9.04) | 9.02 (8.99; 9.04) | 8.96 (8.93; 8.99) |

Data presented as adjusted means. Model 0 was unadjusted. Model 1 was adjusted for age, deprivation index, ethnicity, assessment month, and lifestyle factors (smoking and alcohol intake). Model 2 included additional adjustment for multimorbidity, and Model 3 further adjusted for total energy intake and physical activity.

**Table S5: The association of n-3 fatty acid intake with hand grip strength and muscle mass in older women.**

|  | **Model 0** | **Model 1** | **Model 2** | **Model 3** |
| --- | --- | --- | --- | --- |
| **Grip strength (kg)** | **Mean (95% CI)** | **Mean (95% CI)** | **Mean (95% CI)** | **Mean (95% CI)** |
| n-3 <1.15 g/day | 21.2 (21.1; 21.4) | 21.2 (21.0; 21.4) | 21.2 (21.0; 21.4) | 21.5 (21.3; 21.7) |
| n-3 1.15-1.90 g/day | 21.6 (21.5; 21.7) | 21.6 (21.5; 21.7) | 21.6 (21.5; 21.7) | 21.7 (21.6; 21.8) |
| n-3 1.91-2.65 g/day | 21.8 (21.7; 21.9) | 21.8 (21.7; 22.0) | 21.8 (21.7; 22.0) | 21.9 (21.8; 22.0) |
| n-3 2.66-3.4 g/day | 22.1 (21.9; 22.3) | 22.1 (21.9; 22.3) | 22.1 (21.9; 22.3) | 22.1 (21.9; 22.3) |
| n-3 >3.40 g/day | 22.0 (21.8; 22.2) | 22.0 (21.8; 22.2) | 22.0 (21.8; 22.2) | 21.9 (21.7; 22.1) |
| **Grip Strength Index (kg/m^2^)** |  |  |  |  |
| n-3 <1.15 g/day | 13.1 (13.0; 13.2) | 13.1 (13.0; 13.2) | 13.1 (13.0; 13.2) | 13.3 (13.2; 13.4) |
| n-3 1.15-1.90 g/day | 13.3 (13.2; 13.3) | 13.3 (13.2; 13.3) | 13.3 (13.2; 13.3) | 13.4 (13.3; 31.4) |
| n-3 1.91-2.65 g/day | 13.4 (13.3;13.5) | 13.4 (13.3; 13.5) | 13.4 (13.3; 13.5) | 13.5 (13.4; 13.5) |
| n-3 2.66-3.4 g/day | 13.5 (13.4; 13.6) | 13.5 (13.4; 13.6) | 13.5 (13.4; 13.6) | 13.6 (13.4; 13.7) |
| n-3 >3.40 g/day | 13.5 (13.4; 13.6) | 13.5 (13.4; 13.6) | 13.5 (13.4; 13.6) | 13.5 (13.3; 13.6) |
| **Muscle Mass Index (kg/m^2^)** |  |  |  |  |
| n-3 <1.15 g/day | 6.31 (6.29; 6.33) | 6.31 (6.29; 6.33) | 6.31 (6.29; 6.33) | 6.37 (6.35; 6.40) |
| n-3 1.15-1.90 g/day | 6.38 (6.37; 6.39) | 6.38 (6.37; 6.39) | 6.38 (6.37; 6.39) | 6.41 (6.40; 6.42) |
| n-3 1.91-2.65 g/day | 6.42 (6.41; 6.44) | 6.42 (6.41; 6.44) | 6.42 (6.41; 6.44) | 6.40 (6.38; 6.41) |
| n-3 2.66-3.4 g/day | 6.44 (6.42: 6.46) | 6.44 (6.42; 6.46) | 6.44 (6.42; 6.46) | 6.38 (6.36; 6.40) |
| n-3 >3.40 g/day | 6.44 (6.41; 6.46) | 6.44 (6.41; 6.46) | 6.44 (6.41; 6.46) | 6.37 (6.34; 6.39) |

Data presented as adjusted means. Model 0 was unadjusted. Model 1 was adjusted for age, deprivation index, ethnicity, assessment month, and lifestyle factors (smoking and alcohol intake). Model 2 included additional adjustment for multimorbidity, and Model 3 further adjusted for total energy intake and physical activity.

**Table S6: The association of n-3 fatty acid intake with hand grip strength and muscle mass in older men and women with sarcopenia.**

|  | **Men - Model 3 (n=313)** |  | **Women - Model 3 (n=1,053)** |  |
| --- | --- | --- | --- | --- |
| **Grip strength (kg)** | **Beta (95% CI)** | **p** | **Beta (95% CI)** | **p** |
| n-3 <1.15 g/day | Ref. |  | Ref. |  |
| n-3 1.15-1.90 g/day | 2.793 (0.398; 5.189) | 0.022 | 0.545 (-0.292; 1.383) | 0.202 |
| n-3 1.91-2.65 g/day | 2.784 (0.339; 5.229) | 0.026 | 0.506 (-0.431; 1.444) | 0.290 |
| n-3 2.66-3.4 g/day | 2.008 (-1.782; 4.799) | 0.158 | 0.985 (-0.134; 2.104) | 0.084 |
| n-3 >3.40 g/day | 2.833 (-0.373; 6.040) | 0.083 | 0.490 (-0.895; 1.877) | 0.488 |
| Trend for quintiles | 0.181 (-0. 440; 0. 803) | 0.566 | 0.163 (-0.119; 0.446) | 0.256 |
| **Grip strength Index (kg/m^2^)** |  |  |  |  |
| n-3 <1.15 g/day | Ref. |  | Ref. |  |
| n-3 1.15-1.90 g/day | 1.427 (0.018; 2.836) | 0.047 | 0.363 (-0.156; 0.883) | 0.170 |
| n-3 1.91-2.65 g/day | 1.559 (0.120; 2.997) | 0.034 | 0.353 (-0.228; 0.936) | 0.234 |
| n-3 2.66-3.4 g/day | 1.069 (-0.571; 2.711) | 0.201 | 0.594 (-0.099; 1.289) | 0.093 |
| n-3 >3.40 g/day | 1.640 (-0.245; 3.527) | 0.088 | 0.301 (-0.559; 1.162) | 0.492 |
| Trend for quintiles | 0.141 (-0.223; 0.506) | 0.446 | 0.097 (-0.077; 0.272) | 0.276 |
| **Muscle Mass Index (kg/m^2^)** |  |  |  |  |
| n-3 <1.15 g/day | Ref. |  | Ref. |  |
| n-3 1.15-1.90 g/day | 0.773 (0.228; 1.319) | 0.006 | 0.037 (-0.160; 0.235) | 0.708 |
| n-3 1.91-2.65 g/day | 0.892 (0.336; 1.449) | 0.002 | -0.038 (-0.260; 0.183) | 0.733 |
| n-3 2.66-3.4 g/day | 0.956 (0.320; 1.591) | 0.003 | -0.180 (-0.445; 0.083) | 0.180 |
| n-3 >3.40 g/day | 1.228 (0.498; 1.958) | 0.001 | -0.177 (-0.505; 0.150) | 0.288 |
| Trend for quintiles | 0.197 (0.055; 0.338) | 0.006 | -0.062 (-0.129; 0.004) | 0.068 |

Data presented as adjusted beta-coefficients. Analyses were adjusted for model 3 including age, sex, deprivation index, ethnicity, assessment month, lifestyle factors (smoking and alcohol intake), multimorbidity, total energy intake and physical activity.

**Table S7: The association of n-3 fatty acid intake with hand grip strength and muscle mass in older men and women with sarcopenia.**

|  | **Men - Model 3** | **Women - Model 3** |
| --- | --- | --- |
| **Grip strength (kg)** | **Mean (95% CI)** | **Mean (95% CI)** |
| n-3 <1.15 g/day | 18.8 (16.5; 21.1) | 11.9 (11.2; 12.6) |
| n-3 1.15-1.90 g/day | 21.6 (20.7; 22.5) | 12.5 (12.0; 12.9) |
| n-3 1.91-2.65 g/day | 21.6 (20.7; 22.5) | 12.4 (11.9; 13.0) |
| n-3 2.66-3.4 g/day | 20.8 (19.3; 22.3) | 12.9 (12.1; 13.7) |
| n-3 >3.40 g/day | 21.6 (19.5; 23.8) | 12.4 (11.3; 13.5) |
| **Grip strength Index (kg/m2)** |  |  |
| n-3 <1.15 g/day | 11.0 (9.75; 12.4) | 7.44 (6.99; 7.90) |
| n-3 1.15-1.90 g/day | 12.5 (11.9; 13.0) | 7.81 (7.53; 8.08) |
| n-3 1.91-2.65 g/day | 12.6 (12.1; 13.1) | 7.80 (7.45; 8.14) |
| n-3 2.66-3.4 g/day | 12.1 (11.2; 13.0) | 8.04 (7.55; 8.53) |
| n-3 >3.40 g/day | 12.7 (11.4; 13.9) | 7.74 (7.04; 8.44) |
| **Muscle Mass Index (kg/m2)** |  |  |
| n-3 <1.15 g/day | 8.05 (7.53; 8.56) | 5.97 (5.80; 6.15) |
| n-3 1.15-1.90 g/day | 8.82 (8.61; 9.03) | 6.01 (5.91; 6.12) |
| n-3 1.91-2.65 g/day | 8.94 (8.74; 9.14) | 5.94 (5.80; 6.07) |
| n-3 2.66-3.4 g/day | 9.00 (8.67; 9.34) | 5.79 (5.61; 5.98) |
| n-3 >3.40 g/day | 9.28 (8.79; 9.76) | 5.80 (5.53; 6.06) |

Data presented as adjusted means. Analyses were adjusted for model 3 including age, sex, deprivation index, ethnicity, assessment month, lifestyle factors (smoking and alcohol intake), multimorbidity, total energy intake and physical activity.

**Table S8: The association of the n-6/n-3 fatty acid intake ratio with hand grip strength and muscle mass in older men.**

| **Outcomes** | **Model 0 (n= 27,813)** |  | **Model 1 (n= 27,813)** |  | **Model 2 (n= 27,813)** |  | **Model 3 (n= 27,813)** |  |
| --- | --- | --- | --- | --- | --- | --- | --- | --- |
| **Grip strength (kg)** | **B (95% CI)** | **p** | **B (95% CI)** | **p** | **B (95% CI)** | **p** | **B (95% CI)** | **p** |
| <=2.4 | Ref. |  | Ref. |  | Ref. |  | Ref. |  |
| 2.41-4.8 | 0.323 (-0.270; 0.918) | 0.286 | 0.350 (-0.243; 0.944) | 0.248 | 0.340 (-0.252; 0.933) | 0.260 | 0.137 (-0.484; 0.759) | 0.665 |
| 4.81-7.2 | 0.130 (-0.447; 0.708) | 0.658 | 0.163 (-0.415; 0.741) | 0.581 | 0.169 (-0.406; 0.746) | 0.563 | -0.010 (-0.616; 0.595) | 0.973 |
| 7.21-9.6 | 0.295 (-0.309; 0.899) | 0.339 | 0.322 (-0.281; 0.927) | 0.295 | 0.337 (-0.265; 0.940) | 0.273 | 0.116 (-0.516; 0.750) | 0.718 |
| >9.6 | 0.108 (-0.653; 0.871) | 0.780 | 0.084 (-0.677; 0.847) | 0.827 | 0.072 (-0.687; 0.833) | 0.851 | -0.175 (0.066; 0.678) | 0.667 |
| Trend for quintiles | -0.012 (-0.125; 0.100) | 0.825 | -0.015 (-0.128; 0.097) | 0.792 | -0.008 (-0.121; 0.104) | 0.885 | -0.032 (-0.152; 0.086) | 0.590 |
| **Grip Strength Index (kg/m2)** |  |  |  |  |  |  |  |  |
| <=2.4 | Ref. |  | Ref. |  | Ref. |  | Ref. |  |
| 2.41-4.8 | 0.089 (-0.239; 0.417) | 0.595 | 0.102 (-0.225; 0.431) | 0.540 | 0.097 (-0.229; 0.425) | 0.559 | 0.001 (-0.342; 0.346) | 0.992 |
| 4.81-7.2 | -0.008 (-0.327; 0.311) | 0.960 | 0.007 (-0.311; 0.327) | 0.961 | 0.011 (-0.307; 0.330) | 0.944 | -0.055 (-0.390; 0.280) | 0.747 |
| 7.21-9.6 | 0.096 (-0.237; 0.430) | 0.572 | 0.109 (-0.224; 0.444) | 0.519 | 0.117 (-0.216; 0.450) | 0.491 | 0.029 (-0.321; 0.379) | 0.870 |
| >9.6 | 0.001 (-0.419; 0.422) | 0.994 | -0.012 (-0.433; 0.409) | 0.955 | -0.018 (-0.438; 0.402) | 0.933 | -0.144 (-0.586; 0.297) | 0.523 |
| Trend for quintiles | -0.003 (-0.066; 0.058) | 0.903 | 0.005 (-0.067; 0.057) | 0.864 | -0.002 (-0.064; 0.060) | 0.949 | -0.009 (-0.075; 0.056) | 0.789 |
| **Muscle Mass Index (kg/m2)** |  |  |  |  |  |  |  |  |
| <=2.4 | Ref. |  | Ref. |  | Ref. |  | Ref. |  |
| 2.41-4.8 | -0.038 (-0.099; 0.022) | 0.220 | -0.035 (-0.096; 0.025) | 0.256 | -0.033 (-0.094; 0.026) | 0.272 | -0.081 (-0.144; -0.017) | 0.012 |
| 4.81-7.2 | -0.022 (-0.082; 0.036) | 0.450 | -0.019 (-0.079; 0.039) | 0.512 | -0.020 (-0.079; 0.038) | 0.490 | -0.070 (-0.132; 0.009) | 0.024 |
| 7.21-9.6 | -0.054 (-0.116; 0.007) | 0.084 | -0.052 (0.114; 0.009) | 0.099 | -0.054 (-0.115; 0.007) | 0.085 | -0.104 (-0.168; -0.040) | 0.001 |
| >9.6 | -0.053 (-0.132; 0.024) | 0.177 | -0.058 (-0.136; 0,019) | 0.144 | -0.056 (-0.134; 0.021) | 0.153 | -0.120 (-0.201; -0.38) | 0.004 |
| Trend for quintiles | -0.008 (-0.020; 0.002) | 0.144 | -0.009 (-0.020; 0.002) | 0.117 | -0.010 (-0.021; 0.001) | 0.083 | -0.016 (-0.028; -0.004) | 0.007 |

Data presented as adjusted beta-coefficients. Model 0 was unadjusted. Model 1 was adjusted for age, deprivation index, ethnicity, assessment month, and lifestyle factors (smoking and alcohol intake). Model 2 included additional adjustment for multimorbidity, and Model 3 further adjusted for total energy intake and physical activity.

**Table S9: The association of the n-6/n-3 fatty acid intake ratio with hand grip strength and muscle mass in older women.**

| **Outcomes** | **Model 0 (n=32,041)** |  | **Model 1 (n=32,041)** |  | **Model 2 (n=32,041)** |  | **Model 3 (n=32,041)** |  |
| --- | --- | --- | --- | --- | --- | --- | --- | --- |
| **Grip strength (kg)** | **B (95% CI)** | **p** | **B (95% CI)** | **p** | **B (95% CI)** | **p** | **B (95% CI)** | **p** |
| <=2.4 | Ref. |  | Ref. |  | Ref. |  | Ref. |  |
| 2.41-4.8 | 0.025 (-0.307; 0.357) | 0.882 | 0.029 (-0.302; 0.361) | 0.862 | 0.480 (-0.281; 0.377) | 0.775 | 0.129 (-0.236; 0.495) | 0.489 |
| 4.81-7.2 | -0.221 (-0.545; 0.101) | 0.179 | -0.184 (-0.506; 0.138) | 0.264 | -0.142 (-0.463; 0.178) | 0.383 | -0.088 (-0.445; 0.268) | 0.626 |
| 7.21-9.6 | -0.196 (-0.543; 0.149) | 0.265 | -0.151 (-0.497; 0.194) | 0.392 | -0.128 (-0.473; 0.215) | 0.463 | -0.040 (-0.423; 0.342) | 0.837 |
| >9.6 | -0.247(-0.739; 0.244) | 0.324 | -0.243(-0.734; 0.247) | 0.331 | -0.212 (-0.700; 0.275) | 0.393 | -0.170 (-0.716; 0.375) | 0.540 |
| Trend for quintiles | -0.107 (-0.180; -0.033) | 0.004 | 0.091 (-0.165; -0.018) | 0.014 | -0.085 (-0.158; -0.012) | 0.022 | -0.081 (-0.162; -0.000) | 0.049 |
| **Grip Strength Index (kg/m^2^)** |  |  |  |  |  |  |  |  |
| <=2.4 | Ref. |  | Ref. |  | Ref. |  | Ref. |  |
| 2.41-4.8 | -0.012 (-0.211; 0.187) | 0.094 | -0.010 (-0.209; 0.189) | 0.919 | 0.000 (-0.197; 0.198) | 0.996 | 0.061 (-0.158; 0.282) | 0.581 |
| 4.81-7.2 | -0.143(-0.337; 0.050) | 0.148 | -0.123 (-0.317; 0.070) | 0.211 | -0.100 (-0.293; 0.093) | 0.310 | -0.042 (-0.257; 0.172) | 0.700 |
| 7.21-9.6 | -0.128 (-0.336; 0.080) | 0.228 | -0.103 (-0.311; 0.103) | 0.327 | -0.091 (-0.298; 0.115) | 0.388 | -0.015 (-0.245; 0.215) | 0.898 |
| >9.6 | -0.133 (-0.429; 0.161) | 0.375 | -0.131 (-0.426; 0.164) | 0.384 | -0.113 (-0.407; 0.180) | 0.448 | -0.062 (-0.390; 0.265) | 0.709 |
| Trend for quintiles | -0.057 (-0.101; 0.013) | 0.011 | -0.048 (-0.092; 0.004) | 0.030 | -0.045 (-0.089; -0.001) | 0.043 | -0.036 (-0.085; 0.012) | 0.145 |
| **Muscle Mass Index (kg/m^2^)** |  |  |  |  |  |  |  |  |
| <=2.4 | Ref. |  | Ref. |  | Ref. |  | Ref. |  |
| 2.41-4.8 | 0.019 (-0.021; 0.060) | 0.352 | 0.020 (-0.020; 0.061) | 0.333 | 0.018 (-0.022; 0.059) | 0.376 | -0.021 (0.066; 0.023) | 0.360 |
| 4.81-7.2 | 0.033 (-0.006; 0.073) | 0.103 | 0.032 (-0.007; 0.072) | 0.112 | 0.028 (-0.011; 0.068) | 0.160 | -0.015 (-0.059; 0.028) | 0.480 |
| 7.21-9.6 | 0.026 (-0.016; 0.069) | 0.230 | 0.024 (-0.018; 0.067) | 0.259 | 0.022 (-0.020; 0.065) | 0.300 | -0.019 (-0.061; 0.028) | 0.379 |
| >9.6 | -0.007 (-0.068; 0.053) | 0.809 | -0.008 (-0.069; 0.052) | 0.789 | -0.011 (-0.071; 0.049) | 0.718 | -0.026 (-0.073; 0.020) | 0. 271 |
| Trend for quintiles | 0.003 (-0.005; 0.012) | 0.488 | 0.002 (-0.006; 0.011) | 0.614 | 0.001 (-0.007; 0.010) | 0.706 | -0.005 (-0.015; 0.004) | 0.274 |

Data presented as adjusted beta-coefficients. Model 0 was unadjusted. Model 1 was adjusted for age, deprivation index, ethnicity, assessment month, and lifestyle factors (smoking and alcohol intake). Model 2 included additional adjustment for multimorbidity, and Model 3 further adjusted for total energy intake and physical activity.

**Table S10: The association of the n-6/n-3 fatty acid intake ratio with hand grip strength and muscle mass in older men.**

|  | **Model 0 (n= 27,813)** | **Model 1 (n= 27,813)** | **Model 2 (n= 27,813)** | **Model 3 (n= 27,813)** |
| --- | --- | --- | --- | --- |
| **Grip strength (kg)** | **Mean (95% CI)** | **Mean (95% CI)** | **Mean (95% CI)** | **Mean (95% CI)** |
| <=2.4 | 37.3 (36.7; 37.8) | 36.3 (36.7; 37.8) | 37.3 (36.7; 37.8) | 37.5 (36.9; 38.1) |
| 2.41-4.8 | 37.6 (37.4; 37.8) | 37.6 (37.4; 37.8) | 37.6 (37.4; 37.8) | 37.7 (37.5; 37.8) |
| 4.81-7.2 | 37.4 (37.3; 37.5) | 37.4 (37.3; 37.5) | 37.4 (37.3; 37.5) | 37.5 (37.4; 37.6) |
| 7.21-9.6 | 38.6 (37.4; 38.8) | 37.6 (37.4; 37.8) | 38.6 (37.4; 38.8) | 37.6 (37.4; 37.9) |
| >9.6 | 37.4 (36.9; 37.9) | 37.3 (36.8; 37.9) | 37.3 (36.8; 38.8) | 37.3 (36.8; 37.9) |
| **Grip Strength Index (kg/m^2^)** |  |  |  |  |
| <=2.4 | 21.3 (21.0; 21.6) | 21.3 (21.0; 21.6) | 21.3 (21.0; 21.6) | 21.4 (21.1; 21.7) |
| 2.41-4.8 | 21.4 (21.3; 21.5) | 21.4 (21.3; 21.5) | 21.4 (21.3; 21.5) | 21.4 (21.3; 21.5) |
| 4.81-7.2 | 21.3 (21.2; 21.4) | 21.3 (21.2; 21.4) | 21.3 (21.2; 21.4) | 21.4 (21.3; 21.4) |
| 7.21-9.6 | 21.4 (21.3; 21.5) | 21.4 (21.3; 21.5) | 21.4 (21.3; 21.5) | 21.4 (21.3; 21.6) |
| >9.6 | 21.3 (21.0; 21.6) | 21.3 (21.0; 21.6) | 21.3 (21.0; 21.6) | 21.3 (21.0; 21.6) |
| **Muscle Mass Index (kg/m^2^)** |  |  |  |  |
| <=2.4 | 8.98 (8.92; 9.92) | 8.97 (8.92; 9.03) | 8.97 (8.92; 9.03) | 9.02 (8.92; 8.96) |
| 2.41-4.8 | 8.94 (8.92; 8.96) | 8.94 (8.92; 8.96) | 8.94 (8.92; 8.96) | 8.94 (8.94; 8.96) |
| 4.81-7.2 | 8.95 (8.94; 8.97) | 8.95 (8.94; 8.97) | 8.95 (8.94; 8.97) | 8.95 (8.89; 8.94) |
| 7.21-9.6 | 9.92 (8.90: 8.94) | 8.92 (8.90; 8.94) | 8.92 (8.90; 8.94) | 8.92 (8.90; 8.94) |
| >9.6 | 9.92 (8.87; 8.97) | 8.91 (8.86; 8.97) | 8.92 (8.86; 8.97) | 8.90 (8.85; 8.95) |

Data presented as adjusted means. Model 0 was unadjusted. Model 1 was adjusted for age, deprivation index, ethnicity, assessment month, and lifestyle factors (smoking and alcohol intake). Model 2 included additional adjustment for multimorbidity, and Model 3 further adjusted for total energy intake and physical activity.

**Table S11: The association of the n-6/n-3 fatty acid intake ratio with hand grip strength and muscle mass in older women.**

|  | **Model 0 (n=32,041)** | **Model 1 (n=32,041)** | **Model 2 (n=32,041)** | **Model 3 (n=32,041)** |
| --- | --- | --- | --- | --- |
| **Grip strength (kg)** | **Mean (95% CI)** | **Mean (95% CI)** | **Mean (95% CI)** | **Mean (95% CI)** |
| <=2.4 | 21.8 (21.5; 22.2) | 21.8 (21.5; 22.1) | 21.8 (21.5; 22.1) | 21.8 (21.5; 22.2) |
| 2.41-4.8 | 21.9 (21.8; 22.0) | 21.8 (21.7; 21.0) | 21.8 (21.7; 21.9) | 22.0 (21.8; 22.1) |
| 4.81-7.2 | 21.6 (21.5; 21.7) | 21.6 (21.6; 21.7) | 21.6 (21.6; 21.7) | 21.7 (21.7; 21.8) |
| 7.21-9.6 | 21.6 (21.5; 21.8) | 21.7 (21.5; 21.8) | 21.7 (21.5; 21.8) | 21.8 (21.6; 22.0) |
| >9.6 | 21.6 (21.2; 22.0) | 21.6 (21.2; 22.0) | 21.6 (21.2; 22.0) | 21.7 (21.2; 22.1) |
| **Grip Strength Index (kg/m^2^)** |  |  |  |  |
| <=2.4 | 13.4 (13.3; 13.6) | 13.4 (13.2; 13.6) | 13.4 (13.2; 13.6) | 13.4 (13.2; 13.6) |
| 2.41-4.8 | 13.4 (13.4; 13.5) | 13.4 (13.4; 13.5) | 13.4 (13.3; 13.5) | 13.5 (13.4; 13.6) |
| 4.81-7.2 | 13.3 (13.3; 13.4) | 13.3 (13.3; 13.4) | 13.3 (13.3; 13.4) | 13.4 (13.3; 13.4) |
| 7.21-9.6 | 13.3 (13.2; 13.4) | 13.3 (13.2; 13.4) | 13.3 (13.2; 13.4) | 13.4 (13.3; 13.5) |
| >9.6 | 13.3 (13.1; 13.5) | 13.3 (13.1; 13.5) | 13.3 (13.1; 13.5) | 13.4 (13.1; 13.6) |
| **Muscle Mass Index (kg/m^2^)** |  |  |  |  |
| <=2.4 | 6.37 (6.33; 6.41) | 6.37 (6.33; 6.41) | 6.37 (6.33; 6.41) | 6.41 (6.37; 6.46) |
| 2.41-4.8 | 6.39 (6.37; 6.40) | 6.39 (6.38; 6.40) | 6.39 (6.38; 6.40) | 6.39 (6.38; 6.41) |
| 4.81-7.2 | 6.40 (6.39; 6.41) | 6.40 (6.39; 6.41) | 6.40 (6.39; 6.41) | 6.40 (6.39; 6.41) |
| 7.21-9.6 | 6.40 (6.38; 6.41) | 6.39 (6.38; 6.41) | 6.40 (6.38; 6.41) | 6.39 (6.37; 6.41) |
| >9.6 | 6.36 (6.31; 6.41) | 6.36 (6.31; 6.41) | 6.36 (6.31; 6.41) | 6.36 (6.31; 6.41) |

Data presented as adjusted means. Model 0 was unadjusted. Model 1 was adjusted for age, deprivation index, ethnicity, assessment month, and lifestyle factors (smoking and alcohol intake). Model 2 included additional adjustment for multimorbidity, and Model 3 further adjusted for total energy intake and physical activity.

**Table S12: The association of the n-6/n-3 fatty acid intake ratio with hand grip strength and muscle mass in older men and women with sarcopenia.**

|  | **Men - Model 3 (n=313)** |  | **Women - Model 3 (n=1,053)** |  |
| --- | --- | --- | --- | --- |
| **Grip strength (kg)** | **B (95% CI)** | **p** | **B (95% CI)** | **p** |
| <=2.4 | Ref. |  | Ref. |  |
| 2.41-4.8 | 1.448 (-4.026; 6.924) | 0.603 | -0.336 (-1.954; 1.281) | 0.683 |
| 4.81-7.2 | 1.807 (-3.538; 7.153) | 0.506 | -0.108 (-1.692; 1.476) | 0.893 |
| 7.21-9.6 | 2.403 (-3.025; 7.833) | 0.384 | -0.104 (-1.829; 1.620) | 0.906 |
| >9.6 | 2.238 (-3. 970; 8.448) | 0.478 | -2.618 (-4.791; -0.445) | 0.018 |
| Trend for quintiles | 0.455 (-0. 281; 1. 192) | 0.225 | -0.194 (-0.539; 0.150) | 0.269 |
| **Grip strength Index (kg/m^2^)** |  |  |  |  |
| <=2.4 | Ref. |  | Ref. |  |
| 2.41-4.8 | 0.691 (-2.525; 3.909) | 0.672 | -0.237 (-1.240; 0.765) | 0.642 |
| 4.81-7.2 | 0.786 (-2.354; 3.928) | 0.622 | -0.057 (-1.040; 0.924) | 0.908 |
| 7.21-9.6 | 1.150 (-2.040; 4.340) | 0.478 | -0.094 (-1.164; 0.975) | 0.863 |
| >9.6 | 1.310 (-2.338; 4.959) | 0.480 | -1.719 (-3.067; -0.372) | 0.012 |
| Trend for quintiles | 0.242 (-0.190; 0.675) | 0.271 | -0.125 (-0.339; 0.088) | 0.249 |
| **Muscle Mass Index (kg/m^2^)** |  |  |  |  |
| <=2.4 | Ref. |  | Ref. |  |
| 2.41-4.8 | -0.022 (-1.281; 1.235) | 0.972 | 0.037 (-0.045; 0.721) | 0.084 |
| 4.81-7.2 | 0.073 (-1.155; 1.301) | 0.907 | 0.378 (0.003; 0.753) | 0.048 |
| 7.21-9.6 | -0.200 (-1.448; 1.047) | 0.752 | 0.266 (-0.142; 0.675) | 0.201 |
| >9.6 | 0.296 (-1.130; 1.723) | 0.683 | -0.005 (-0.519; 0.509) | 0.984 |
| Trend for quintiles | -0.025 (-0.196; 0.144) | 0.765 | -0.017 (-0.099; 0.064) | 0.674 |

Data presented as adjusted beta coefficients. Analyses were adjusted for model 3 including age, sex, deprivation index, ethnicity, assessment month, lifestyle factors (smoking and alcohol intake), multimorbidity, total energy intake and physical activity.

**Table S13: The association of the n-6/n-3 fatty acid intake ratio with hand grip strength and muscle mass in older men and women with sarcopenia.**

|  | **Men - Model 3 (n=313)** | **Women - Model 3 (n=1,053)** |
| --- | --- | --- |
| **Grip strength (kg)** | **Mean (95% CI)** | **Mean (95% CI)** |
| <=2.4 | 19.5 (14.2; 24.8) | 12.7 (11.1; 14.2) |
| 2.41-4.8 | 20.9 (19.7; 22.1) | 12.3 (11.8; 12.8) |
| 4.81-7.2 | 21.3 (20.6; 22.0) | 12.6 (12.2; 12.9) |
| 7.21-9.6 | 21.9 (20.6; 23.2) | 12.9 (11.8; 13.3) |
| >9.6 | 21.7 (18.4; 25.0) | 10.0 (8.56; 11.6) |
| **Grip strength Index (kg/m^2^)** |  |  |
| <=2.4 | 11.6 (8.48; 14.7) | 7.94 (6.99; 8.89) |
| 2.41-4.8 | 12.2 (11.5; 12.9) | 7.70 (7.38; 8.02) |
| 4.81-7.2 | 12.3 (11.9; 12.8) | 7.88 (7.64; 8.12) |
| 7.21-9.6 | 12.7 (11.9; 13.5) | 7.84 (7.36; 8.32) |
| >9.6 | 12.9 (10.9; 14.8) | 6.22 (5.27; 7.17) |
| **Muscle Mass Index (kg/m^2^)** |  |  |
| <=2.4 | 8.86 (7.65; 10.0) | 5.62 (5.25; 5.98) |
| 2.41-4.8 | 8.84 (8.57; 9.12) | 5.95 (5.83; 6.08) |
| 4.81-7.2 | 8.94 (8.77; 9.10) | 6.00 (5.90; 6.09) |
| 7.21-9.6 | 8.66 (8.36; 8.96) | 5.88 (5.70; 6.07) |
| >9.6 | 9.16 (8.40; 9.92) | 5.61 (5.25; 5.98) |

Data presented as adjusted means. Analyses were adjusted for model 3 including age, sex, deprivation index, ethnicity, assessment month, lifestyle factors (smoking and alcohol intake), multimorbidity, total energy intake and physical activity.

**Table S14: The association of n-3 fatty acid intake with hand grip strength and muscle mass in older men and women aged ≥65.**

|  | **Men (n=12,254)**  **n-3 fatty acid**  **intake**  **Model 3** |  | **Men (n=171)**  **n-3 fatty acid**  **intake with sarcopenia**  **Model 3** |  | **Women (n=12,457)**  **n-3 fatty acid**  **intake**  **Model 3** |  | **Women (n=519)**  **n-3 fatty acid**  **intake**  **with sarcopenia Model 3** |  |
| --- | --- | --- | --- | --- | --- | --- | --- | --- |
| **Grip strength (kg)** | **B (95% CI)** | **p** | **B (95% CI)** | **p** | **B (95% CI)** | **p** | **B (95% CI)** | **p** |
| n-3 <1.15 g/day | Ref. |  | Ref. |  | Ref. |  | Ref. |  |
| n-3 1.15-1.90 g/day | 0.292 (-0.278; 0.863) | 0.315 | 3.643 (-0.052; 7.339) | 0.053 | 0.131 (-0.215; 0.477) | 0.459 | 0.583 (-0.484; 1.651) | 0.283 |
| n-3 1.91-2.65 g/day | 0.596 (0.001; 1.193) | 0.051 | 4.342 (0.537; 8.147) | 0.026 | 0.259 ( -0.118;0.637) | 0.178 | 0.128 ( -1.068;1.325) | 0.833 |
| n-3 2.66-3.4 g/day | 0.641 (-0.023;1.307) | 0.059 | 2.165 (-2.014; 6.345) | 0.307 | 0.497 (0.048; 0.946) | 0.030 | 0.855 (-0.608; 2.318) | 0.251 |
| n-3 >3.40 g/day | 0.236 (-0.472; 0.495) | 0.513 | 2.330 (-2.190; 6.851) | 0.310 | 0.278 (-0.215; 0.773) | 0.269 | 0.492 (-0.608; 2.318) | 0.611 |
| Trend for quintiles | 0.072 (-0.067; 0.213) | 0.309 | -0.081(-0.940; 0.777) | 0.851 | 0.102 (-0.001; 0.206) | 0.053 | 0.106 (-0.268; 0.480) | 0.577 |
| **Grip strength Index (kg/m^2^)** |  |  |  |  |  |  |  |  |
| n-3 <1.15 g/day | Ref. |  | Ref. |  | Ref. |  | Ref. |  |
| n-3 1.15-1.90 g/day | 0.093 (-0.224;0.410) | 0.565 | 1.896 (-0.256;4.049) | 0.084 | 0.064 (-0.145;0.274) | 0.547 | 0.393 (-0.266;1.054) | 0.242 |
| n-3 1.91-2.65 g/day | 0.241 (-0.090;0.574) | 0.154 | 2.455 (0.238;4.671) | 0.030 | 0.124 (-0.103;0.353) | 0.284 | 0.169 (-0.570;0.909) | 0.653 |
| n-3 2.66-3.4 g/day | 0.244 (-0.125;0.614) | 0.195 | 1.201 (-1.233;3.636) | 0.331 | 0.262 (-0.009;0.533) | 0.058 | 0.539 (-0.365;1.444) | 0.242 |
| n-3 >3.40 g/day | 0.078 (-0.315;0.472) | 0.698 | 1.309 (-1.324;3.942) | 0.327 | 0.150 (-0.148;0.449) | 0.323 | 0.335 (-0.843;1.513) | 0.577 |
| Trend for quintiles | 0.030 (-0.047;0.108) | 0.448 | -0.004 (-0.503;0.494) | 0.986 | 0.032 (-0.008;0.117) | 0.087 | 0.075 (-0.156;0.306) | 0.523 |
| **Muscle Mass Index (kg/m^2^)** |  |  |  |  |  |  |  |  |
| n-3 <1.15 g/day | Ref. |  | Ref. |  | Ref. |  | Ref. |  |
| n-3 1.15-1.90 g/day | 0.369 (-0.233; 0.097) | 0.230 | 0.338 (-0.455; 1.131) | 0.401 | 0.049 (0.005;0.092) | 0.027 | 0.067 (-0.198;0.333) | 0.617 |
| n-3 1.91-2.65 g/day | 0.030 (-0.032; 0.093) | 0.342 | 0.808 (-0.008; 1.625) | 0.052 | 0.039 (-0.008;0.086) | 0.104 | 0.088 (-0.209;0.386) | 0.561 |
| n-3 2.66-3.4 g/day | 0.048 (-0.021; 0.119) | 0.173 | 0.398 (-0.499; 1.295) | 0.382 | 0.033 (-0.022;0.090) | 0.238 | -0.150 (-0.515;0.213) | 0.416 |
| n-3 >3.40 g/day | 0.044 (-0.030; 0.119) | 0.248 | 0.911 (-0.059; 1.881) | 0.066 | 0.021 (-0.040;0.083) | 0.504 | -0.096 (-0.571;0.377) | 0.689 |
| Trend for quintiles | 0.007 (-0.007; 0.021) | 0.364 | 0.169 (-0.013; 0.351) | 0.070 | 0.000 (-0.012;0.013) | 0.933 | -0.037 (-0.130;0.055) | 0.430 |

Data presented as adjusted beta coefficients. Model 0 was unadjusted. Model 1 was adjusted for age, deprivation index, ethnicity, assessment month, and lifestyle factors (smoking and alcohol intake). Model 2 included additional adjustment for multimorbidity, and Model 3 further adjusted for total energy intake and physical activity.

**Table S15: The association of n-3 fatty acid intake with hand grip strength and muscle mass in older men and women aged ≥65 with and without sarcopenia.**

|  | **Men (n=12,254)**  **n-3 fatty acid**  **Model 3** | **Men (n=171)**  **n-3 fatty acid**  **with sarcopenia - Model 3** | **Women (n=12,457)**  **n-3 fatty acid**  **Model 3** | **Women (n=519)**  **n-3 fatty acid**  **with sarcopenia - Model 3** |
| --- | --- | --- | --- | --- |
| **Grip strength (kg)** | **Mean (95% CI)** | **Mean (95% CI)** | **Mean (95% CI)** | **Mean (95% CI)** |
| n-3 <1.15 g/day | 36.0 (35.4; 36.5) | 17.6 (14.1; 21.1) | 20.8 (20.5; 21.1) | 12.1 (11.1; 13.0) |
| n-3 1.15-1.90 g/day | 36.2 (36.0; 36.5) | 21.2 (19.9; 22.5) | 20.9 (20.8; 21.1) | 12.6 (12.1; 13.2) |
| n-3 1.91-2.65 g/day | 36.5 (36.3; 36.8) | 21.9 (20.5; 23.3) | 21.1 (20.8; 21.3) | 12.2 (11.5; 12.9) |
| n-3 2.66-3.4 g/day | 36.6 (36.2; 37.0) | 19.7 (17.5; 22.0) | 21.3 (21.0; 21.6) | 12.9 (11.9; 14.0) |
| n-3 >3.40 g/day | 36.2 (35.8; 36.6) | 19.9 (17.1; 22.7) | 21.1 (20.7; 21.4) | 12.6 (10.9; 14.2) |
| **Grip strength Index (kg/m^2^)** |  |  |  |  |
| n-3 <1.15 g/day | 20.6 (20.3; 20.9) | 10.4 (8.38; 12.4) | 12.8 (12.7; 13.0) | 7.53 (6.95; 8.10) |
| n-3 1.15-1.90 g/day | 20.7 (20.6; 20.8) | 12.3 (11.5; 13.0) | 12.9 (12.8; 13.0) | 7.92 (7.56; 8.27) |
| n-3 1.91-2.65 g/day | 20.9 (20.7; 21.0) | 12.8 (12.0; 13.6) | 13.0 (12.8; 13.1) | 7.69 (7.25; 8.14) |
| n-3 2.66-3.4 g/day | 20.9 (20.7; 21.1) | 11.6 (10.3; 12.9) | 13.1 (12.9; 13.3) | 8.06 (7.41; 8.72) |
| n-3 >3.40 g/day | 20.7 (20.5; 20.9) | 11.7 (10.1; 13.3) | 13.0 (12.8; 13.2) | 7.86 (6.86; 8.86) |
| **Muscle Mass Index (kg/m^2^)** |  |  |  |  |
| n-3 <1.15 g/day | 8.86 (8.80; 8.91) | 8.25 (7.51; 9.00) | 6.30 (6.26; 6.34) | 5.90 (5.67; 6.13) |
| n-3 1.15-1.90 g/day | 8.89 (8.87; 8.92) | 8.59 (8.31; 8.88) | 6.35 (6.32; 6.37) | 5.96 (5.82; 6.11) |
| n-3 1.91-2.65 g/day | 8.89 (8.86; 8.91) | 9.06 (8.77; 9.36) | 6.34 (6.31; 6.36) | 5.98 (5.80; 6.17) |
| n-3 2.66-3.4 g/day | 8.91 (8.87; 8.94) | 8.65 (8.17; 9.13) | 6.33 (6.29; 6.37) | 5.75 (5.48; 6.01) |
| n-3 >3.40 g/day | 8.90 (8.86; 8.95) | 9.16 (8.57; 9.76) | 6.32 (6.27; 6.36) | 5.80 (5.40; 6.20) |

Data presented as adjusted means. Analyses were adjusted for model 3 including age, sex, deprivation index, ethnicity, assessment month, lifestyle factors (smoking and alcohol intake), multimorbidity, total energy intake and physical activity.

**Table S16: The association of the n-6/n-3 fatty acid intake ratio with hand grip strength and muscle mass in older men and women aged ≥65 with and without sarcopenia.**

|  | **Men (n= 12,057)**  **n-6/n-3 ratio**  **Model 3** |  | **Men (n=171)**  **n-6/n-3 ratio with sarcopenia**  **Model 3** |  | **Women (n= 11,888)**  **n-6/n-3 ratio**  **Model 3** |  | **Women (n=519)**  **n-6/n-3 ratio with sarcopenia**  **Model 3** |  |
| --- | --- | --- | --- | --- | --- | --- | --- | --- |
| **Grip strength (kg)** | **B (95% CI)** | **p** | **B (95% CI)** | **p** | **B (95% CI)** | **p** | **B (95% CI)** | **p** |
| <=2.4 | Ref. |  | Ref. |  | Ref. |  | Ref. |  |
| 2.41-4.8 | -0.197 (-1.069; 0.674) | 0.657 | 3.368 (-3.176; 9.912) | 0.310 | 0.116 (-0.453;0.685) | 0.689 | 0.705 (-1.308; 2.719) | 0.491 |
| 4.81-7.2 | -0.221 (-1.069; 0.626) | 0.608 | 3.338 (-2.903; 9.580) | 0.292 | -0.128 (-0.684;0.427) | 0.650 | 0.471 (-1.503; 2.447) | 0.639 |
| 7.21-9.6 | -0.091 (-0.986; 0.804) | 0.842 | 2.430 (-3.979; 8.841) | 0.454 | 0.147 (-0.455;0.750) | 0.631 | 0.288 (-1.904; 2.481) | 0.796 |
| >9.6 | -0.959 (-2.142; 0.223) | 0.112 | 4.316 (-3.518; 12.151) | 0.278 | -0.164 (-1.033;0.704) | 0.711 | -1.467 (-4.404;1.469) | 0.327 |
| Trend for quintiles | -0.057 (-0. 234;0.118) | 0.521 | 0.059 (-0.963; 1.083) | 0.908 | -0.033 (-0.164;0.097) | 0.621 | -0.264 (-0.724;0.194) | 0.258 |
| **Grip strength Index (kg/m^2^)** |  |  |  |  |  |  |  |  |
| <=2.4 | Ref. |  | Ref. |  | Ref. |  | Ref. |  |
| 2.41-4.8 | -0.180 (-0.665; 0.303) | 0.464 | 1.669 (-2.136; 5.475) | 0.387 | 0.037 (-0.306;0.382) | 0.829 | 0.448 (-0.795;1.693) | 0.479 |
| 4.81-7.2 | -0.179 (-0.651; 0.291) | 0.455 | 1.608 (-2.021; 5.238) | 0.382 | -0.090 (-0.427;0.245) | 0.597 | 0.318 (-1.192;1.518) | 0.608 |
| 7.21-9.6 | -0.107 (-0.604; 0.390) | 0.673 | 1.139 (-2.588; 4.867) | 0.546 | 0.081 (-0.282;0.446) | 0.659 | 0.162 (-1.192;1.518) | 0.813 |
| >9.6 | -0.585 (-1.242; 0.728) | 0.081 | 2.537 (-2.019; 7.093) | 0.273 | -0.079 (-0.604;0.446) | 0.768 | -0.945 (-2.760;0.870) | 0.307 |
| Trend for quintiles | -0.031 (-0.129; 0.669) | 0.530 | 0.043 (-0.551; 0.638) | 0.885 | -0.010 (-0.089;0.068) | 0.800 | -0.170 (-0.454;0.113) | 0.237 |
| **Muscle Mass Index (kg/m^2^)** |  |  |  |  |  |  |  |  |
| <=2.4 | Ref. |  | Ref. |  | Ref. |  | Ref. |  |
| 2.41-4.8 | -0.111 (-0.203; -0.019) | 0.017 | -0.186 (-1.557;1.184) | 0.788 | 0.014 (-0.056;0.086) | 0.683 | 0.401 (-0.100;0.903) | 0.117 |
| 4.81-7.2 | -0.074 (-0.164; 0.014) | 0.101 | -0.275 (-1.583;1.032) | 0.677 | 0.019 (-0.050;0.088) | 0.588 | 0.342 (-0.150;0.834) | 0.173 |
| 7.21-9.6 | -0.139 (-0.234; -0.045) | 0.004 | -0.814 (-2.157;0.529) | 0.233 | 0.004 (-0.071;0.079) | 0.910 | 0.215 (-0.331;0.761) | 0.439 |
| >9.6 | -0.094 (-0.219; 0.030) | 0.137 | 0.573 (-1.068;2.215) | 0.491 | 0.001 (-0.110;0.107) | 0.981 | 0.322 (-0.409;1.055) | 0.386 |
| Trend for quintiles | -0.013 (-0.032; 0.005) | 0.158 | -0.128 (-0.347;0.091) | 0.249 | -0.001(-0.018;0.014) | 0.825 | -0.017 (-0.131;0.097) | 0.770 |

Data presented as adjusted beta coefficients. Model 0 was unadjusted. Model 1 was adjusted for age, deprivation index, ethnicity, assessment month, and lifestyle factors (smoking and alcohol intake). Model 2 included additional adjustment for multimorbidity, and Model 3 further adjusted for total energy intake and physical activity.

**Table S17: The association of the n-6/n-3 fatty acid intake ratio with hand grip strength and muscle mass in older men and women aged ≥65 with and without sarcopenia.**

|  | **Men (n= 12,057)**  **n-6/n-3 ratio**  **Model 3** | **Men (n=171)**  **n-6/n-3 with sarcopenia - Model 3** | **Women (n= 11,888)**  **n-6/n-3 ratio**  **Model 3** | **Women (n=519)**  **n-6/n-3 ratio with sarcopenia**  **Model 3** |
| --- | --- | --- | --- | --- |
| **Grip strength (kg)** | **Mean (95% CI)** | **Mean (95% CI)** | **Mean (95% CI)** | **Mean (95% CI)** |
| <=2.4 | 36.6 (35.7; 37.4) | 17.8 (11.6; 23.9) | 21.0 (20.5; 21.5) | 12.0 (10.1; 13.9) |
| 2.41-4.8 | 36.4 (36.1; 36.7) | 21.1 (19.5; 22.7) | 21.1 (20.9; 21.3) | 12.7 (12.1; 13.4) |
| 4.81-7.2 | 36.3 (36.2; 36.5) | 21.1 (20.0; 22.3) | 20.9 (20.7; 21.0) | 12.5 (12.0; 13.0) |
| 7.21-9.6 | 36.5 (36.1; 36.8) | 20.2 (18.2; 22.2) | 21.2 (20.9; 21.4) | 12.3 (11.2; 13.4) |
| >9.6 | 35.6 (34.8; 36.5) | 22.1 (17.1; 27.1) | 20.9 (20.2; 21.5) | 10.5 (8.34; 12.8) |
| **Grip strength Index (kg/m^2^)** |  |  |  |  |
| <=2.4 | 20.9 (20.5; 21.4) | 10.7 (7.12; 14.2) | 13.0 (12.7; 13.3) | 7.52 (6.34; 8.69) |
| 2.41-4.8 | 20.8 (20.6; 20.9) | 12.3 (11.4; 13.3) | 13.0 (12.9; 13.1) | 7.97 (7.56; 8.37) |
| 4.81-7.2 | 20.8 (20.7; 20.9) | 12.3 (11.6; 12.9) | 12.9 (12.8; 13.0) | 7.83 (7.52; 8.14) |
| 7.21-9.6 | 20.8 (20.6; 21.0) | 11.8 (10.6; 13.0) | 13.1 (12.9; 13.2) | 8.68 (7.02; 8.34) |
| >9.6 | 20.4 (19.9; 20.8) | 13.2 (10.3; 16.1) | 12.9 (12.5; 13.3) | 7.57 (5.18; 7.96) |
| **Muscle Mass Index (kg/m^2^)** |  |  |  |  |
| <=2.4 | 8.98 (8.90; 9.07) | 9.10 (7.81; 10.3) | 6.32 (6.25; 6.38) | 5.59 (5.11; 6.06) |
| 2.41-4.8 | 8.87 (8.84; 8.90) | 8.92 (8.58; 9.25) | 6.33 (6.31; 6.36) | 5.99 (5.83; 6.15) |
| 4.81-7.2 | 8.91 (8.89; 8.93) | 8.83 (8.59; 9.07) | 6.34 (6.32; 6.36) | 5.93 (5.81; 6.06) |
| 7.21-9.6 | 8.85 (8.81; 8.88) | 8.29 (7.86; 8.71) | 6.32 (6.29; 6.36) | 5.80 (5.54; 6.07) |
| >9.6 | 9.89 (8.80; 9.98) | 9.68 (8.62; 10.7) | 6.32 (6.23; 6.40) | 5.91 (5.35; 6.47) |

Data presented as adjusted means. Analyses were adjusted for model 3 including age, sex, deprivation index, ethnicity, assessment month, lifestyle factors (smoking and alcohol intake), multimorbidity, total energy intake and physical activity.
